# Supplementary material for: Effectiveness of interventions for people living with dementia and their carers in Chinese communities: protocol for a systematic review and meta-analysis of randomised controlled trials
Source: BMJ Open. 2021 Aug 17;11(8):e047560. doi: 10.1136/bmjopen-2020-047560 (PMC8372806; doi:10.1136/bmjopen-2020-047560)
Supplement: Author's manuscript [file bmjopen-2020-047560.draft_revisions.pdf]

BMJ Open is committed to open peer review. As part of this commitment we make the peer review history of every article we publish publicly available.

When an article is published we post the peer reviewers' comments and the authors' responses online. We also post the versions of the paper that were used during peer review. These are the versions that the peer review comments apply to.

The versions of the paper that follow are the versions that were submitted during the peer review process. They are not the versions of record or the final published versions. They should not be cited or distributed as the published version of this manuscript.

BMJ Open is an open access journal and the full, final, typeset and author-corrected version of record of the manuscript is available on our site with no access controls, subscription charges or pay-per-view fees (http://bmjopen.bmj.com).

If you have any questions on BMJ Open's open peer review process please email info.bmjopen@bmj.com

BMJ Open

Effectiveness of interventions for people living with dementia and their carers in Chinese Communities: protocol for a systematic review and meta-analysis of randomised controlled trials

Journal:	*BMJ Open*
Manuscript ID	bmjopen-2020-047560
Article Type:	Protocol
Date Submitted by the Author:	02-Dec-2020

Complete List of Authors:	Shi, Cheng; University of Hong Kong, Department of Social Work and Social Administration; Beijing Normal University, Center for Social Welfare Studies<br>Chen, Shuangzhou; University of Hong Kong, Department of Social Work and Social Administration<br>Salcher-Konrad, Maximilian; LSE, Health Policy<br>Choy, Jacky CP ; University of Hong Kong, Department of Social Work and Social Administration<br>Luo, Hao; Hong Kong University,<br>Leung, Dara Kiu Yi; Hong Kong University, Social Work and Social Administration<br>Cai, Xinxin; University of Hong Kong, Department of Social Work and Social Administration<br>Zeng, Yue; University of Hong Kong, Department of Social Work and Social Administration<br>Dai, Ruizhi; University of Hong Kong<br>Comas-Herrera, Adelina; London School of Economics and Political Science, Care Policy and Evaluation Centre; CPEC, London School of Economics and Political Sci<br>Knapp, Martin; London School of Economics, Personal Social Services Research Unit<br>McDaid, David ; London School of Economics<br>Wong, Gloria; Hong Kong University,

Keywords:	Dementia < NEUROLOGY, Old age psychiatry < PSYCHIATRY, PUBLIC HEALTH, Health policy < HEALTH SERVICES ADMINISTRATION & MANAGEMENT

SCHOLARONE™
Manuscripts

I, the Submitting Author has the right to grant and does grant on behalf of all authors of the Work (as defined in the below author licence), an exclusive licence and/or a non-exclusive licence for contributions from authors who are: i) UK Crown employees; ii) where BMJ has agreed a CC-BY licence shall apply, and/or iii) in accordance with the terms applicable for US Federal Government officers or employees acting as part of their official duties; on a worldwide, perpetual, irrevocable, royalty-free basis to BMJ Publishing Group Ltd ("BMJ") its licensees and where the relevant Journal is co-owned by BMJ to the co-owners of the Journal, to publish the Work in this journal and any other BMJ products and to exploit all rights, as set out in our licence.

The Submitting Author accepts and understands that any supply made under these terms is made by BMJ to the Submitting Author unless you are acting as an employee on behalf of your employer or a postgraduate student of an affiliated institution which is paying any applicable article publishing charge ("APC") for Open Access articles. Where the Submitting Author wishes to make the Work available on an Open Access basis (and intends to pay the relevant APC), the terms of reuse of such Open Access shall be governed by a Creative Commons licence – details of these licences and which Creative Commons licence will apply to this Work are set out in our licence referred to above.

Other than as permitted in any relevant BMJ Author's Self Archiving Policies, I confirm this Work has not been accepted for publication elsewhere, is not being considered for publication elsewhere and does not duplicate material already published. I confirm all authors consent to publication of this Work and authorise the granting of this licence.

For peer review only

Title page

Effectiveness of interventions for people living with dementia and their carers in Chinese Communities: protocol for a systematic review and meta-analysis of randomised controlled trials

Cheng Shi[1,2], Shuangzhou Chen[1], Maximilian Salcher-Konrad[3], Jacky CP Choy[1], Hao Luo,[1,4] Dara KY Leung[1], Xinxin Cai[1], Yue Zeng[1], Ruizhi Dai[5], Adelina Comas-Herrera[3], David McDaid[3], Martin Knapp[3], Gloria HY Wong[1]

Correspondence to Gloria Hoi Yan Wong, 524 The Jockey Club Tower, Centennial Campus, The University of Hong Kong, Pokfulam, Hong Kong. Email: ghywong@hku.hk

Author affiliations

[1]Department of Social Work and Social Administration, The University of Hong Kong, Hong Kong

[2]Center for Social Welfare Studies, Beijing Normal University, Beijing, China

[3]Care Policy and Evaluation Centre, London School of Economics and Political Science, London, UK

[4]Department of Computer Science, The University of Hong Kong, Hong Kong

[5]Faculty of Social Sciences, The University of Hong Kong, Hong Kong

Author Contributions

GHYW, CS, SC, JCPC, ACH and MK defined the scope of the review and review question. GHYW, CS, SC, and MSK developed inclusion/exclusion criteria. HL designed the method for Chinese database search. GHYW, CS, SC, MSK, and DMD consulted on the databases to be searched and search terms used. GHYW, CS, SC, MSK, JCPC, and DKYL provided information

For peer review only - http://bmjopen.bmj.com/site/about/guidelines.xhtml

on methods of data synthesis. CS drafted the protocol. All authors contributed to the study design and reviewed the draft protocol. GHYW is the guarantor of the manuscript.

Funding This work is conducted as part of the "Tools to Inform Policy: Chinese Communities Actions in Response to Dementia" (TIP-CARD) project, supported by Hong Kong Research Impact Fund of the Research Grants Council (Project Reference No.: R7017-18). MSK, DMD and ACH's contribution was supported by the UK Research and Innovation's Global Challenges Research Fund (ES/P010938/1) as part of the Strengthening Responses to Dementia in Developing Countries project (STRiDE). The funders were not involved in the development of this protocol.

Competing interests None declared

Patient consent for publication Not required.

Provenance and peer review Not commissioned; externally previewed.

Word Count: 2,901/4,000

For peer review only - http://bmjopen.bmj.com/site/about/guidelines.xhtml

Effectiveness of interventions for people living with dementia and their carers in Chinese Communities: protocol for a systematic review and meta-analysis of randomised controlled trials

ABSTRACT

Introduction: As the largest and rapidly ageing population, Chinese are now the major driver of the continued growth in dementia prevalence globally. The need for evidence-based intervention and care services in Chinese Communities is urgent. A wide range of pharmacological and non-pharmacological interventions for dementia have been trialled in Chinese populations, although the evidence has not been systemically reviewed. This systematic review and meta-analysis aims to synthesise and compare the evidence on the effectiveness of these interventions for people living with dementia and their carers in Chinese communities worldwide.

Methods and analysis: This protocol follows the Preferred Reporting Items for Systematic Review and Meta-Analysis Protocols checklist. Chinese electronic databases (China National Knowledge Infrastructure, WanFang DATA) and English electronic databases (MEDLINE, EMBASE, PsycINFO, CINAHL Plus, Global Health, WHO Global Index Medicus, Virtual Health Library, Cochrane CENTRAL, Social Care Online, BASE, MODEM Toolkit, Cochrane Database of Systematic Reviews) will be searched. Hand searching of reference lists will complement database searches. We will include studies that evaluated the effectiveness of interventions for dementia or mild cognitive impairment in Chinese populations using a randomised control trial design, published between January 2008 and June 2020. We will use a standardised form to extract data and version 2 of the Cochrane risk-of-bias tool for randomized trials to assess the risk of bias of the included studies. Collected data will be fully interpreted with narrative synthesis and

For peer review only - http://bmjopen.bmj.com/site/about/guidelines.xhtml

analysed using pairwise and network meta-analysis to pool intervention effects. We will perform subgroup analysis and meta-regression to explore the potential reasons for heterogeneity.

Ethics and dissemination: No formal ethics approval is required for this protocol. The findings will facilitate the development of studies on interventions for dementia and timely inform dementia policymaking and practice. Planned dissemination channels include peer-reviewed publication, conference presentations and websites.

PROSPERO registration number: CRD42019134135

Strengths and Limitations of this study

- This systematic review and meta-analysis will be the first review of randomised control trials on effectiveness of both pharmacological and non-pharmacological interventions for people living with dementia and their carers in Chinese communities worldwide.
- We will use a comprehensive search strategy of publications in both Chinese electronic databases (China National Knowledge Infrastructure, WanFang DATA) and English electronic databases (MEDLINE, EMBASE, PsycINFO, CINAHL Plus, Global Health, WHO Global Index Medicus, Virtual Health Library, Cochrane CENTRAL, Social Care Online, BASE, MODEM Toolkit, Cochrane Database of Systematic Reviews)
- We will narratively synthesise the collected data to map the interventions studied in Chinese communities, and conduct pairwise and network meta-analysis to compare the effectiveness of interventions.
- This review will be limited to the number and quality of trials conducted in Chinese communities.

For peer review only - http://bmjopen.bmj.com/site/about/guidelines.xhtml

INTRODUCTION

Around 50 million people currently live with dementia worldwide, of whom 20 per cent are Chinese populations.[1] Chinese populations refer to people of Chinese ethnicity or national heritage regardless of their nationality and region of residence. As the largest and rapidly ageing population, Chinese are now the major driver in the continued growth of global dementia prevalence.[2] Due to the physical and emotional challenges in caring, dementia not only affects people living with the condition, but also their families, formal carers and other supporters.[3] With a culture emphasising filial piety, coupled with insufficient care services, family care is often the main supporting resource for people living with dementia (PLwD) in Chinese communities worldwide. Dementia has been recognised as one of the most burdensome diseases among Chinese populations .[4]

There is currently no cure for dementia, although symptoms can be managed with effective intervention and good care.[3] China has recently launched its national dementia strategy; one of the main tasks is to improve the wellbeing of PLwD by increasing service provision.[5] Taiwan has updated its dementia policy in 2017, with promoting dementia research, innovation, and development being one of its seven strategies.[6] In Macau's 10-year Plan of Action on Dementia Services published in 2016, strengthening community services and caregiver support are one of its five strategies.[7] In Hong Kong, a government services review and programme plan published in 2017[8 9] highlighted the need to strengthen services for PLwD and recommended a seven-stage model for dementia service in accordance with the World Health Organization and Alzheimer's Disease International's framework. The need for evidence-based intervention and care services in Chinese populations is urgent.

For peer review only - http://bmjopen.bmj.com/site/about/guidelines.xhtml

Research on dementia interventions appears to be sparse in Asian populations.[2] Most evidence on drug treatment and non-pharmacological interventions came from Western countries, with questionable relevance for Chinese populations. For example, Cognitive Stimulation Therapy (CST) used alone or in combination with medication was shown to be effective and even cost-effective in improving cognition and quality of life,[10-13] leading to a recommendation for routine use in England's National Institute for Health and Care Excellence (NICE),[14] and by Alzheimer's Disease International.[15] In contrast, preliminary findings from a study applying CST in Hong Kong Chinese suggest that a larger number of participants needed to treat to achieve clinically significant improvement in cognition.[16] Such discrepancies in an intervention's effect, possibly due to cultural differences, highlight the importance of generating evidence relevant to local populations.

There is now increasing evidence on a wide range of interventions for dementia undertaken in Chinese populations. A few reviews have been published, focusing on specific interventions and subtypes of dementia, such as the efficacy of donepezil in Chinese with Alzheimer's disease,[17] Chinese herbal medicine as adjunctive therapy for vascular dementia,[18] and traditional Chinese mind-body exercise (baduanjin) in older adults with mild cognitive impairment (MCI).[19] Growing evidence also suggests that the therapeutic response to dementia intervention (e.g., donepezil) might differ between Chinese and Western populations due to pharmacogenetic factors,[17 20] which calls for more accurate estimates of interventions tailored to Chinese populations.[21]

There are ongoing studies aiming to synthesise evidence for dementia intervention and care, including the Modelling Outcome and Cost Impacts of Interventions for Dementia (MODEM) project[22] with a dementia evidence toolkit (https://www.modem-dementia.org.uk/) covering dementia interventions in English literature, and the Strengthening Responses to Dementia in Developing countries (STRiDE; https://stride-dementia.org/) project with an ongoing systematic

For peer review only - http://bmjopen.bmj.com/site/about/guidelines.xhtml

review and meta-analysis on the evidence in low- and middle-income countries.[23] For Chinese populations, there lacks a comprehensive evidence synthesis on the effectiveness of dementia or dementia-related interventions that cover different types of dementia (e.g. Alzheimer's disease, vascular dementia, frontotemporal dementia, Lewy body disease, mixed dementia and MCI) and interventions (e.g. pharmacological treatment, psychosocial intervention, and traditional Chinese medicine). Existing systematic reviews have focused mainly on the English language literature, where evidence from high-income areas such as Hong Kong and Taiwan can be found. Although Chinese academic databases have been recognised as valuable resource for dementia-related studies, they have not been fully explored.[24-26]

This systematic review and meta-analysis aims to comprehensively synthesise the evidence in Chinese and English to identify interventions for PLwD and their carers in Chinese populations, as part of a research project, Tools to Inform Policy: Chinese Communities Actions in Response to Dementia (TIP-CARD; www.tip-card.hku.hk/) that aligns with the above-mentioned dementia evidence synthesis effort in low- and middle-income countries.[23]

METHODS AND ANALYSIS

Protocol and registration

This protocol for the systematic review and meta-analysis followed the Preferred Reporting Items for Systematic Review and Meta-Analysis Protocols checklist.[27] This study has been registered on PROSPERO (www.crd.york.ac.uk/prospero; registration number: CRD42019134135).

Eligible criteria

For peer review only - http://bmjopen.bmj.com/site/about/guidelines.xhtml

Population

We will include studies conducted among adults (aged 18 years and over) living with dementia or MCI and their carers in Chinese populations.

For studies on PLwD, we will include studies covering people living with any type and stage of dementia. Dementia or major neurocognitive disorder includes but is not limited to Alzheimer's disease, vascular dementia, frontotemporal dementia, Lewy body disease, and mixed dementia, and MCI or mild neurocognitive disorder. People with MCI will be included due to their higher risk of developing dementia in later years,[28] as well as vascular cognitive impairment, no dementia (VCIND). Studies conducted in people with diseases co-occurring with dementia will also be included. We will exclude studies without explicating the diagnostic criteria for dementia or MCI when recruiting participants.

Dementia carers include everyone providing care, regardless of whether or not they are paid, and whether or not they are living with the person they care for.

We focus on studies conducted among people of Chinese ethnicity or national heritage regardless of their nationality and region of residence. Studies with less than 50% of Chinese participants or where no results are available for a Chinese subgroup will be excluded.

Intervention

Based on the effectiveness perspective,[23 29] any type of interventions for improving desired outcomes will be eligible. We will include studies on pharmacological treatment, non-pharmacological intervention (e.g., cognitive intervention, technological intervention, training, and exercise) or multicomponent interventions. We will exclude studies: 1) where no clear

For peer review only
For peer review only - http://bmjopen.bmj.com/site/about/guidelines.xhtml

intervention was described, 2) on primary prevention of dementia, and 3) on non-interventional studies.

Comparison

Any type of comparisons will be eligible for inclusion, such as active comparators, treatment as usual, placebo, and no treatment.

Outcomes

Any type of outcomes of dementia intervention will be eligible for inclusion, which may affect individuals, family, dementia care workforce, wider society, and social or health care systems. As people with dementia often have complex problems with symptoms in many domains,[21] previous review studies on dementia interventions have measured outcomes including cognition, neuropsychiatric symptoms, functioning, quality of life, and carer burden.[30-32] We will extract measures of outcomes and results in all data forms as reported in included studies.

Study design

To identify likely causal relationships, we will only include studies using randomized controlled trials (RCT) or cluster RCT designs. To control study quality, we will only include RCTs with a low risk of bias in the process of evidence generation. According to version 2 of the Cochrane risk-of-bias tool for randomized trials (RoB 2) [33], random allocation sequence should be generated by methods with low risk of bias, such as computer-generated random numbers, a random number table, coin tossing, shuffling cards or envelopes, throwing dice, or drawing lots. Studies using no

For peer review only - http://bmjopen.bmj.com/site/about/guidelines.xhtml

random element or providing no information on the generation process of the random allocation sequence will be excluded.

To minimise small-study effects,[34 35] we will exclude the studies with a sample size of less than 50 in either the intervention group or comparison group(s).

Publication type

We will include the primary publications of intervention studies and grey literature as long as they have evaluated the effectiveness of dementia interventions in Chinese populations. Relevant systematic reviews or scoping reviews will be included in the first step screening, and then will be used to complement the primary publications by a manual search of the reference lists. Conference abstracts will be included only if they contain sufficient information to assess eligibility for inclusion.

Publication period

Studies published between January 2008 and June 2020.

Language

Studies will be limited to English and Chinese language publications.

Information sources

For peer review only - http://bmjopen.bmj.com/site/about/guidelines.xhtml

We will search two major Chinese electronic databases (China National Knowledge Infrastructure and WanFang DATA) and English electronic databases (MEDLINE, EMBASE, PsycINFO, CINAHL Plus, Global Health, WHO Global Index Medicus, Virtual Health Library, Cochrane CENTRAL, Social Care Online, BASE, MODEM Toolkit, Cochrane Database of Systematic Reviews). Hand searching of reference lists among review studies will complement the database searches.

Search strategy

We adapted an established search strategy protocol[23] used to search for English language literature. Corresponding Chinese search terms were translated and adapted by three bilingual researchers (GHYW, SC, and CS) experienced in dementia/ageing research with a training background in psychology, psychiatry, translation, social work and social policy from Hong Kong and mainland China. Search terms in English and Chinese are listed in Table 1. In studies published in English, the search terms related to Chinese populations include "China", "Chinese", "Sino", "Hong Kong", "Taiwan", "Taiwanese", "Macau" and "Asian".

For studies published in English, we will first extract eligible study records identified from an ongoing systematic review[23], which used same search strategy and search terms for dementia intervention and covered studies published between 2008 and 2018. Then we will search the terms on Chinese population in title and abstract. Second, we will repeat the search process in English databases mentioned above to identify studies published between 2019 and 2020.

For studies published in Chinese, we will use Python,[36] a programming language, to facilitate Chinese database searching by using dementia-related search terms (search items #1-4 in Table 1). The reason is the technical challenge posed by limitations on the number of search terms

For peer review only - http://bmjopen.bmj.com/site/about/guidelines.xhtml

and exported records per time in the two Chinese databases. The searching results for dementia-related study records will be exported in an Excel spreadsheet. Then we will search the intervention-related terms (search items #5-53) in the title and abstract.

Study records

Data management

To deal with a potentially large number of search results and the variety of data source in two languages, references will be managed using two web-based software packages during the review and extraction process. First, literature search results will be uploaded to Rayyan (https://rayyan.qcri.org/), a web and mobile app that can facilitate the initial screening of abstracts and titles using a semi-automated process.[37] Then, we will use Covidence (https://www.covidence.org/), an Internet-based software for managing systematic reviews, for full-text review, risk of bias assessment and data extraction. Both Rayyan and Covidence record the reasons for excluding study records at each level.

Duplicated publications will be checked based on title, author, journal, and year using Excel, as well as using the find duplicates function in Rayyan and Covidence. Multiple publications from the same study will be identified based on the key information (e.g. juxtaposing authors names, studies design, intervention, and outcomes) from the full-texts, or by contacting authors for clarification if needed. Once confirmed, the included multiple publications will be linked on Covidence.

Study selection

For peer review only - http://bmjopen.bmj.com/site/about/guidelines.xhtml

Study selection will be a two-step process, with detailed explanations for inclusion and exclusion criteria in each step. First, two researchers will independently screen the title and abstract and make a decision of inclusion or exclusion. A justification (criterion) will be required for any exclusion decision. Studies with insufficient information in title and abstract for making a decision will be included at this stage. The Rayyan machine learning-based classifier[37] will be considered to facilitate the title and abstract screening given the potentially overloading work. Using a certain number of manually screened studies as a training dataset, Rayyan will generate a relevance rating for each study, ranging from 0.5 (lowest) to 5 (highest).[37] We may use a low relevance score (e.g., below 1.5) as a threshold to guide study exclusion.

Second, the included studies after title and abstract screening will be uploaded to Covidence for full-text review. Two independent reviewers will review the full text of each study and make a decision, with a justification provided for each excluded study. Review studies will be excluded at this step, although their reference lists will be used to comlement the database search results.

All disagreements in each step will be resolved through discussion between the two reviewers. If consensus is unreachable, a third reviewer will be consulted for a final decision.

Upon completion of the selection process, we will generate a Preferred Reporting Items for Systematic Reviews and Meta-Analyses flow chart[38] to illustrate the inclusion and exclusion of studies at each stage in study selection.

Data collection process

We will use a standardised form based on the template available from Covidence for data extraction. The form will be pilot-tested using included studies. To ensure data consistency across

For peer review only - http://bmjopen.bmj.com/site/about/guidelines.xhtml

reviewers, we will organise exercises and group discussions as trainings of reviewers. Due to the anticipated large number of potentially eligible studies, one reviewer will complete the data extraction form with verification by the other reviewer. We will keep all records of corrections or amendments to the data extraction. In studies where the needed information is not reported, we will contact the authors to request for information.

Data items

We will extract information on items listed in Box 1 from the included studies.

Outcomes and prioritisation

The effectiveness perspective will be used when evaluating dementia interventions. For PLwD, our primary interest will be the changes in cognition after treatment. For carers, we will primarily focus on changes in quality of life. We will also evaluate other common outcomes such as changes in functional level in PLwD and carer burden if sufficient information is available for analysis.

Risk of bias in individual studies

We will use Cochrane Collaboration's the recently updated risk of bias tool (RoB 2)[33] to assess the quality of included studies in Covidence. Two reviewer will independently make judgements based on the criteria for judging the risk of bias. Disagreement will be resolved by discussion and arbitrated by a third reviewer if consensus is unreachable.

Data synthesis

For peer review only - http://bmjopen.bmj.com/site/about/guidelines.xhtml

Evidence on dementia-related interventions in PLwDs and carers will be analysed separately. Studies of family-based or dyadic interventions involving both PLwDs and carers will be categorised according to the subject of each outcome.

Qualitative analysis

We will first describe and summarise disease characteristics, features of the intervention, number of participants, a summary of participant characteristics, outcomes, and indication of risk of bias assessment in a tabular form. Then, we will undertake a narrative synthesis by grouping data based on sample characteristics and types of interventions (e.g., pharmacological treatment, traditional Chinese medicine, and non-pharmacological interventions). We will map the available evidence to identify the research priorities and gaps.

Quantitative synthesis

Where sufficient information is available, we will conduct meta-analyses to examine the effect of interventions on the outcomes of interest using aggregated data. First, we will measure the effect size of the expected intervention outcomes for each study. Effect measures will depend on the type of outcome data. For example, standard means will be used to measure the effect size if a continuous scale is used for changes in cognition. Second, individual study results will be combined to give an overall summary estimate using a random-effects model to pool the study statistics of each study as a default, considering both within- and between-study variability. We will also run a fixed-effects model to test the robustness of model choice.

For peer review only - http://bmjopen.bmj.com/site/about/guidelines.xhtml

We will perform pairwise meta-analysis to compare the effectiveness of the same two interventions.[39] For comparing the effectiveness of multiple interventions, we will use network meta-analysis, to combine direct and indirect evidence among various studies.[40]

Sensitivity analyses will be conducted to explore the robustness of the meta-analysis results by varying the analytic data or methods.[41] For example, we will perform sensitivity analyses for studies with a low risk of bias and trials using placebo as a comparator.

Subgroup analysis and Meta-regression

We will calculate the Cochran's Q statistic and the I^2 statistic to estimate heterogeneity of the included studies.[42] If statistical heterogeneity is observed, we will conduct subgroup analysis and meta-regression to explore the potential reasons for the differences. Potential candidate covariates for subgroup analysis and meta-regression include intervention characteristics (e.g., types of intervention, intervention dosage and duration), participant characteristics (e.g., age, gender, education, severity of dementia, and type of dementia), care settings and locations (e.g., mainland China, Hong Kong, Taiwan, Macau, and other Chinese communities worldwide).

Meta-bias(es)

We will use funnel plots to detect meta-biases. Statistical tests will be performed for funnel plot asymmetry, which can indicate the presence of publication bias and selective outcome reporting.

Confidence in cumulative estimate

For peer review only - http://bmjopen.bmj.com/site/about/guidelines.xhtml

We will use the Grading of Recommendations Assessment, Development and Evaluation (GRADE) approach[43] to assess the quality of evidence. The domains of the assessment include risk of bias, inconsistency, indirectness of evidence, imprecision, and publication bias.

Patient and public involvement

Patients and the public will not be involved in the design or development of this review protocol. However, stakeholders including PLwD, family members, care staff, healthcare professionals and policymakers will be engaged in dissemination plan as described below.

ETHICS AND DISSEMINATION

This protocol for a systematic review and meta-analysis describes the methods to identify and synthesize published evidence on the effectiveness of interventions for PLwD and their carers in Chinese communities. No formal ethics approval is required for this protocol. The findings from this review will facilitate the development of studies on interventions for dementia and timely inform dementia policymaking and practice. Planned dissemination channels include peer-reviewed publication, conference presentations and publicly accessible websites.

For peer review only

For peer review only - http://bmjopen.bmj.com/site/about/guidelines.xhtml

REFERENCES

1. Patterson C. World Alzheimer report 2018: the state of the art of dementia research: new frontiers. Alzheimer's Disease International (ADI), London, 2018.

2. Prince M, Wimo A, Guerchet M, et al. World Alzheimer Report 2015：The Global Impact of Dementia. Alzheimer's Disease International (ADI), London, 2015.

3. Livingston G, Sommerlad A, Orgeta V, et al. Dementia prevention, intervention, and care. *Lancet* 2017;390(10113):2673-734. doi: 10.1016/S0140-6736(17)31363-6

4. Lou VWQ, Chi I. Is China Ready For Dementia. *Journal of Alzheimers & Neurodegenerative Diseases* 2020:6: 041. doi: 10.24966/AND-9608/100041

5. National Health Commission. Work Plan on Exploring special services for senile dementia prevention and treatment (Tansuo laonian chidai fangzhi tese fuwu gongzuo fang'an), 2020.

6. Ministry of Health and Welfare, Republic of China (Taiwan),. Taiwan Dementia Policy: A Framework for Prevention and Care 2.0, 2017.

7. Government of Macao SAR. 10-Year Plan of Action on Services for Older Persons 2016-2025. In: SAR GoM, ed., 2016.

8. Food and Health Bureau. Mental Health Review Report. Hong Kong: Food and Health Bureau, Hong Kong Special Administrative Region Government, 2017.

9. Working Group on Elderly Services Programme Plan, Elderly Commission of Government of Hong Kong SAR,. Elderly Services Programme Plan, 2017.

10. Woods B, Aguirre E, Spector AE, et al. Cognitive stimulation to improve cognitive functioning in people with dementia. *Cochrane Database Syst Rev* 2012;2:CD005562. doi: 10.1002/14651858.CD005562.pub2

For peer review only

11. Huntley JD, Gould RL, Liu K, et al. Do cognitive interventions improve general cognition in dementia? A meta-analysis and meta-regression. *BMJ Open* 2015;5(4):e005247. doi: 10.1136/bmjopen-2014-005247 [published Online First: 2015/04/04]

12. Knapp M, Thorgrimsen L, Patel A, et al. Cognitive stimulation therapy for people with dementia: cost-effectiveness analysis. *The British journal of psychiatry : the journal of mental science* 2006;188:574-80. doi: 10.1192/bjp.bp.105.010561 [published Online First: 2006/06/02]

13. D'Amico F, Rehill A, Knapp M, et al. Maintenance cognitive stimulation therapy: an economic evaluation within a randomized controlled trial. *J Am Med Dir Assoc* 2015;16(1):63-70. doi: 10.1016/j.jamda.2014.10.020 [published Online First: 2014/12/22]

14. National Institute for Health and Care Excellence. Dementia: assessment, management and support for people living with dementia and their carers: NICE 2018.

15. International AsD. World Alzheimer Report 2011: The benefits of early diagnosis and intervention: Alzheimer’s Disease International London, 2011.

16. Wong GHY, Yek OPL, Zhang AY, et al. Cultural adaptation of cognitive stimulation therapy (CST) for Chinese people with dementia: multicentre pilot study. *International journal of geriatric psychiatry* 2017 doi: 10.1002/gps.4663

17. Zhang N, Gordon ML. Clinical efficacy and safety of donepezil in the treatment of Alzheimer's disease in Chinese patients. *Clin Interv Aging* 2018;13:1963-70. doi: 10.2147/CIA.S159920

18. Zeng LF, Zou YP, Kong LS, et al. Can Chinese Herbal Medicine Adjunctive Therapy Improve Outcomes of Senile Vascular Dementia? Systematic Review with Meta-analysis of Clinical Trials. *Phytother Res* 2015;29(12):1843-57. doi: 10.1002/ptr.5481

For peer review only

For peer review only - http://bmjopen.bmj.com/site/about/guidelines.xhtml

19. Xia R, Qiu PT, Lin HY, et al. The Effect of Traditional Chinese Mind-Body Exercise (Baduanjin) and Brisk Walking on the Dorsal Attention Network in Older Adults With Mild Cognitive Impairment. *Front Psychol* 2019;10 doi: ARTN 2075

10.3389/fpsyg.2019.02075

20. Liu HC, Hong CJ, Wang SJ, et al. ApoE genotype in relation to AD and cholesterol - A study of 2,326 Chinese adults. *Neurology* 1999;53(5):962-66. doi: Doi 10.1212/Wnl.53.5.962

21. Livingston G, Huntley J, Sommerlad A, et al. Dementia prevention, intervention, and care: 2020 report of the Lancet Commission. *Lancet* 2020;396(10248):413-46. doi: 10.1016/S0140-6736(20)30367-6

22. Comas-Herrera A, Knapp M, Wittenberg R, et al. MODEM: A comprehensive approach to modelling outcome and costs impacts of interventions for dementia. Protocol paper. *BMC Health Serv Res* 2017;17(1):25. doi: 10.1186/s12913-016-1945-x

23. Salcher-Konrad M, Naci H, McDaid D, et al. Effectiveness of interventions for dementia in low- and middle-income countries: protocol for a systematic review, pairwise and network meta-analysis. *BMJ Open* 2019;9(6):e027851. doi: 10.1136/bmjopen-2018-027851

24. Fung ICH. Chinese journals: a guide for epidemiologists. *Emerg Themes Epidemi* 2008;5 doi: Artn 2010.1186/1742-7622-5-20

25. Xia J, Wright J, Adams CE. Five large Chinese biomedical bibliographic databases: accessibility and coverage. *Health Info Libr J* 2008;25(1):55-61. doi: 10.1111/j.1471-1842.2007.00734.x

26. Chan KY, Wang W, Wu JJ, et al. Epidemiology of Alzheimer's disease and other forms of dementia in China, 1990-2010: a systematic review and analysis. *Lancet* 2013;381(9882):2016-23. doi: 10.1016/S0140-6736(13)60221-4

For peer review only - http://bmjopen.bmj.com/site/about/guidelines.xhtml

27. Shamseer L, Moher D, Clarke M, et al. Preferred reporting items for systematic review and meta-analysis protocols (PRISMA-P) 2015: elaboration and explanation. *BMJ* 2015;350:g7647. doi: 10.1136/bmj.g7647 [published Online First: 2015/01/04]

28. Mitchell AJ, Shiri-Feshki M. Rate of progression of mild cognitive impairment to dementia– meta-analysis of 41 robust inception cohort studies. *Acta Psychiatrica Scandinavica* 2009;119(4):252-65.

29. Reeves BC, Deeks JJ, Higgins J. 13 Including non-randomized studies. *Cochrane handbook for systematic reviews of interventions* 2008;1:391.

30. Chien WT, Lee IYM. Randomized controlled trial of a dementia care programme for families of home-resided older people with dementia. *J Adv Nurs* 2011;67(4):774-87. doi: 10.1111/j.1365-2648.2010.05537.x

31. Lam LCW, Lee JSW, Chung JCC, et al. A randomized controlled trial to examine the effectiveness of case management model for community dwelling older persons with mild dementia in Hong Kong. *Int J Geriatr Psych* 2010;25(4):395-402. doi: 10.1002/gps.2352

32. Young DKW, Ng PYN, Kwok T, et al. The effects of an expanded cognitive stimulation therapy model on the improvement of cognitive ability of elderly with mild stage Dementia living in a community - a randomized waitlist controlled trial. *Aging Ment Health* 2019;23(7):855-62. doi: 10.1080/13607863.2018.1471586

33. Sterne JA, Savović J, Page MJ, et al. RoB 2: a revised tool for assessing risk of bias in randomised trials. *bmj* 2019;366 doi: https://doi.org/10.1136/bmj.l4898

34. Chen HY, Shen DT, Ji DZ, et al. Prevalence and burden of hepatitis D virus infection in the global population: a systematic review and meta-analysis. *Gut* 2019;68(3):512-21. doi: 10.1136/gutjnl-2018-316601 [published Online First: 2018/09/20]

For peer review only - http://bmjopen.bmj.com/site/about/guidelines.xhtml

For peer review only

35. Turner RM, Bird SM, Higgins JP. The impact of study size on meta-analyses: examination of underpowered studies in Cochrane reviews. *PLoS One* 2013;8(3):e59202. doi: 10.1371/journal.pone.0059202 [published Online First: 2013/04/02]

36. Foundation PS. Python language reference, version 3.6. 8. 2020

37. Ouzzani M, Hammady H, Fedorowicz Z, et al. Rayyan—a web and mobile app for systematic reviews. *Systematic Reviews* 2016;5(1):210. doi: 10.1186/s13643-016-0384-4

38. Moher D, Liberati A, Tetzlaff J, et al. Preferred Reporting Items for Systematic Reviews and Meta-Analyses: The PRISMA Statement. *PLOS Medicine* 2009;6(7):e1000097. doi: 10.1371/journal.pmed.1000097

39. Lau J, Ioannidis JP, Schmid CH. Quantitative synthesis in systematic reviews. *Ann Intern Med* 1997;127(9):820-6. doi: 10.7326/0003-4819-127-9-199711010-00008 [published Online First: 1998/02/12]

40. Cuijpers P, Noma H, Karyotaki E, et al. Effectiveness and Acceptability of Cognitive Behavior Therapy Delivery Formats in Adults With Depression: A Network Meta-analysis. *JAMA Psychiatry* 2019;76(7):700-07. doi: 10.1001/jamapsychiatry.2019.0268

41. Centre for Reviews and Dissemination. CRD's guidance for undertaking reviews in healthcare: CRD, University of York 2009.

42. Higgins JP, Thompson SG. Quantifying heterogeneity in a meta-analysis. *Stat Med* 2002;21(11):1539-58. doi: 10.1002/sim.1186 [published Online First: 2002/07/12]

43. Schünemann H, Brożek J, Guyatt G, et al. Handbook for grading the quality of evidence and the strength of recommendations using the GRADE approach: Cochrane Collaboration; 2013 [Available from: https://gdt.gradepro.org/app/handbook/handbook.html accessed 19 Oct 2020.

For peer review only

For peer review only - http://bmjopen.bmj.com/site/about/guidelines.xhtml

TABLES

Table 1 Search terms related to dementia and intervention in English and Simplified Chinese

Search #	Search terms in English	Search terms in Simplified Chinese
1	Dementia	痴呆 or 失智 or 认知症
2	Cognitive disorder	认知障碍 or 认知功能障碍 or 认知紊乱 or 认知功能紊乱
3	Alzheimer	茨海默 or 兹海默
4	((cognit* or memory or cerebr*) adj3 (impair* or los* or declin* or deteriorat* or degenerat*)).mp.	(认知 or 记忆 or 脑) (缺损 or 缺失 or 退*化 or 衰退 or 下降 or 损伤 or 恶化 or 损害 or 退行)
5	(Intervention* or therap* or treatment* or program* or manage* or prevent* or diagnos* or polic*).mp.	干预 or 介入 or 治疗 or 疗法 or 方案 or 处理 or 预防 or 诊断 or 措施 or 手段 or 政策 or 应用 or 支持 or 效果 or 疗效 or 观察 or 价值 or 临床 or 分析
6	Cognitive therapy	认知 (治疗 or 疗法)
7	Cognitive stimulation	认知 (刺激 or 促进)
8	Cognitive training	认知训练
9	Cognitive rehabilitation	认知 (复康 or 复健 or 康复)
10	Drug therapy or pharmacotherapy	*药*
11	Cholinesterase inhibitors	胆碱分解抑制剂 or 胆碱酵素抑制剂 or 胆碱酶抑制剂
12	Cholinesterase agent	胆碱分解剂 or 胆碱酵素剂 or 胆碱酶剂
13	(Sedative or tranquili* adj3 (agent* or drug*)).mp.	(镇静 or 镇定 or 安神 or 安定) (药 or 剂)
14	Antipsychotic or neuroleptic (agent* or drug*)	抗精神病 (药 or 剂)
15	exp Serotonin Reuptake Inhibitors or ssri	(血清素 or 5-羟色胺) (再摄取 or 再吸收 or 回收) 抑制剂
16	Benzodiazepines	苯二氮平 or 苯二氮卓
17	(memantine or donepezil or rivastigmine or galantamine or souvenaid or risperidone or haloperidol or olanzapine	(美金刚 or 美金胺) (多奈哌齐 or 多奈哌其) (卡巴拉汀 or 利斯的明) (加兰他敏 or 加兰他明 or 格兰他明) (智敏捷) (利培酮 or 利螺环酮) (氟哌啶醇 or 氟哌丁苯 or 氟哌醇 or 卤吡醇) (奥氮平) (喹硫平) (西酞普兰) (右美沙芬 or 右旋美沙酚 or 右旋美索芬 or 右甲吗喃) (卡馬西

For peer review only - http://bmjopen.bmj.com/site/about/guidelines.xhtml

	or quetiapine or citalopram or dextromethorphan or carbamazepine or mirtazapine or sertraline or moclobemide or trazodone or melatonin or ramelteon or methylphenidate).mp.	平 or 卡马平 or 卡巴氮平 or 卡巴马平) (米氮平) (舍曲林) (吗氯贝胺) (曲唑酮) (褪黑素 or 褪黑激素) (雷美替胺 or 拉米替隆) (哌甲酯 or 派醋甲酯 or 盐酸甲酯)
18	Movement Therapy	(运动 or 动作)
19	(Physical activit* or physical training).mp.	(运动 or 体育 or 体能) (活动 or 训练)
20	(social adj3 activit*).mp.	社交活动 or 社会活动
21	Psychotherapy	心理 (治疗 or 疗法)
22	(behavio?r* adj3 therap*).mp.	行为 (治疗 or 疗法)
23	Counseling	辅导 or 咨询
24	((Psychosocial or psycho social) adj3 (support or interven* or care)).ti,ab.	（社会心理 or 社交心理）（支援 or 治疗 or 干预 or 介入 or 照顾)
25	Alternative medicine	(替代 or 另类) (治疗 or 疗法 or 医学 or 医疗)
26	Chinese medicine	中医 or 中药
27	Acupuncture	针灸 or 针刺 or 电针
28	(herb* adj3 (tea or remedy or remedies or medicine*)).ti,ab.	草药 or 药草 or 药用植物 or 草本 or 茶疗
29	Gingko	银杏 or 白果
30	homeopathy	(顺势 or 同质 or 同种)
31	((music or art or aroma or light or photo or pet or pets) adj3 therap*).ti,ab.	(音乐 or 艺术 or 香薰 or 光照 or 光线 or 宠物 or 动物 or 舞蹈)
32	Massage	按摩 or 推拿
33	Mind Body Therapy	身心 or 心身 or 正念 or 冥想
34	Advance directives	预设医疗指示 or 预设指示 or 预前意愿 or 预先指示
35	(Advance? adj3 (care or medical or healthcare) adj3 plan*).mp.	(预设 or 预立) (护理计划 or 临终*计划 or 医疗决定)
36	(decision* adj3 (aid* or support)).mp.	决策援助 or 决策辅助 or 决策支持
37	Case Management	个案管理
38	(communicati* adj3 skill* adj3 training).mp.	沟通技巧 (训练 or 培训)

For peer review only - http://bmjopen.bmj.com/site/about/guidelines.xhtml

39	(dementia care adj3 map*).mp.	认知障碍症照顾图谱 or 老年痴呆症照顾图谱 or 失智症照顾测绘
40	((person* or patient*) adj3 cent* adj3 care).mp.	(以人为本 or 人本 or 以人为中心 or 病人为本) (照顾 or 照护 or 护理 or 治疗 or 医疗)
41	((caregiver or carer) adj3 educat*).mp.	(照顾者 or 家属 or 家庭 or 照护者 or 照料者) 教育
42	Support Groups	（支援 or 支持 or 互助）（小组 or 组）
43	Self-Help Techniques	自助法 or 自助*法 or 自助技巧 or 自救*
44	Social Support	社交支援/社交支持/互助组
45	Computer assisted diagnosis	电脑辅助诊断/计算机辅助诊断
46	Telemedicine	远程医疗 or 远距医疗 or 远距离医疗
47	Computer Assisted Therapy	电脑辅助治疗 or 计算机辅助治疗
48	Mobile Devices	移动设备 or 行动装置
49	((smart adj2 (phone* or device* or tablet*)) or smartphone*).mp.	智能 (手机 or 电话 or 平板电脑) or 智能* or 可穿戴*
50	cognitive aid	认知辅助/认知帮助
51	Reminder	提示 or 提醒
52	Robot	机器人
53	Animal Experiment	动物实验/动物试验

Box 1 Data to be extracted from included studies

General information
- Reviewer who extracting the data
- Date of data extraction
- Publication details and identification
- Research site: places (city-level) where the trial was conducted
- Care setting

Study characteristics
- Study design
- Study inclusion and exclusion criteria
- Recruitment procedures

Participants characteristics
- Demographics (e.g., age, gender)
- Socio-economic status (e.g., education)
- Clinical features (e.g., types of dementia, severity and duration of dementia)

Intervention
- Description of the intervention(s) and comparator(s) (e.g., name, treatment dose, duration, components and how it was delivered).
- Intervention types (e.g., pharmaceutical interventions, traditional Chinese medicine, non-pharmacological treatment and multicomponent interventions).

Outcomes and results
- Outcomes: outcome list and how each outcome was measured (instruments used)
- Results: effect size and measure of its variance for the outcomes of interest

Risk of bias information
- Judgements based on the criteria of the RoB 2.

For peer review only - http://bmjopen.bmj.com/site/about/guidelines.xhtml

Reporting checklist for protocol of a systematic review.

Based on the PRISMA-P guidelines.

Instructions to authors

Complete this checklist by entering the page numbers from your manuscript where readers will find each of the items listed below.

Your article may not currently address all the items on the checklist. Please modify your text to include the missing information. If you are certain that an item does not apply, please write "n/a" and provide a short explanation.

Upload your completed checklist as an extra file when you submit to a journal.

In your methods section, say that you used the PRISMA-Preporting guidelines, and cite them as:

Moher D, Shamseer L, Clarke M, Ghersi D, Liberati A, Petticrew M, Shekelle P, Stewart LA. Preferred Reporting Items for Systematic Review and Meta-Analysis Protocols (PRISMA-P) 2015 statement. Syst Rev. 2015;4(1):1.

		Reporting Item	Page Number
**Title**			
Identification	#1a	Identify the report as a protocol of a systematic review	1
Update	#1b	If the protocol is for an update of a previous systematic review, identify as such	n/a
**Registration**			
	#2	If registered, provide the name of the registry (such as PROSPERO) and registration number	4
**Authors**			
Contact	#3a	Provide name, institutional affiliation, e-mail address of all protocol authors; provide physical mailing address of corresponding author	1
Contribution	#3b	Describe contributions of protocol authors and identify the	1-2

For peer review only

		guarantor of the review	
**Amendments**			
	#4	If the protocol represents an amendment of a previously completed or published protocol, identify as such and list changes; otherwise, state plan for documenting important protocol amendments	n/a
**Support**			
Sources	#5a	Indicate sources of financial or other support for the review	2
Sponsor	#5b	Provide name for the review funder and / or sponsor	2
Role of sponsor or funder	#5c	Describe roles of funder(s), sponsor(s), and / or institution(s), if any, in developing the protocol	2
**Introduction**			
Rationale	#6	Describe the rationale for the review in the context of what is already known	5-7
Objectives	#7	Provide an explicit statement of the question(s) the review will address with reference to participants, interventions, comparators, and outcomes (PICO)	7
**Methods**			
Eligibility criteria	#8	Specify the study characteristics (such as PICO, study design, setting, time frame) and report characteristics (such as years considered, language, publication status) to be used as criteria for eligibility for the review	7-10
Information sources	#9	Describe all intended information sources (such as electronic databases, contact with study authors, trial registers or other grey literature sources) with planned dates of coverage	10-11
Search strategy	#10	Present draft of search strategy to be used for at least one electronic database, including planned limits, such that it could be repeated	11
Study records - data management	#11a	Describe the mechanism(s) that will be used to manage records and data throughout the review	12
Study records -	#11b	State the process that will be used for selecting studies (such	12-13

For peer review only

selection process		as two independent reviewers) through each phase of the review (that is, screening, eligibility and inclusion in meta-analysis)	
Study records - data collection process	#11c	Describe planned method of extracting data from reports (such as piloting forms, done independently, in duplicate), any processes for obtaining and confirming data from investigators	13
Data items	#12	List and define all variables for which data will be sought (such as PICO items, funding sources), any pre-planned data assumptions and simplifications	14
Outcomes and prioritization	#13	List and define all outcomes for which data will be sought, including prioritization of main and additional outcomes, with rationale	14
Risk of bias in individual studies	#14	Describe anticipated methods for assessing risk of bias of individual studies, including whether this will be done at the outcome or study level, or both; state how this information will be used in data synthesis	14
Data synthesis	#15a	Describe criteria under which study data will be quantitatively synthesised	15
Data synthesis	#15b	If data are appropriate for quantitative synthesis, describe planned summary measures, methods of handling data and methods of combining data from studies, including any planned exploration of consistency (such as I2, Kendall's τ)	15
Data synthesis	#15c	Describe any proposed additional analyses (such as sensitivity or subgroup analyses, meta-regression)	15-16
Data synthesis	#15d	If quantitative synthesis is not appropriate, describe the type of summary planned	14-15
Meta-bias(es)	#16	Specify any planned assessment of meta-bias(es) (such as publication bias across studies, selective reporting within studies)	16
Confidence in cumulative evidence	#17	Describe how the strength of the body of evidence will be assessed (such as GRADE)	16

For peer review only

The PRISMA-P checklist is distributed under the terms of the Creative Commons Attribution License CC-BY 4.0. This checklist was completed on 02. December 2020 using https://www.goodreports.org/, a tool made by the EQUATOR Network in collaboration with Penelope.ai

For peer review only

BMJ Open

Effectiveness of interventions for people living with dementia and their carers in Chinese communities: protocol for a systematic review and meta-analysis of randomised controlled trials

Journal:	*BMJ Open*
Manuscript ID	bmjopen-2020-047560.R1
Article Type:	Protocol
Date Submitted by the Author:	04-Jun-2021
Complete List of Authors:	Shi, Cheng; University of Hong Kong, Department of Social Work and Social Administration; Beijing Normal University, Center for Social Welfare Studies<br>Chen, Shuangzhou; University of Hong Kong, Department of Social Work and Social Administration<br>Salcher-Konrad, Maximilian; LSE, Health Policy<br>Choy, Jacky CP ; University of Hong Kong, Department of Social Work and Social Administration<br>Luo, Hao; Hong Kong University,<br>Leung, Dara Kiu Yi; Hong Kong University, Social Work and Social Administration<br>Cai, Xinxin; University of Hong Kong, Department of Social Work and Social Administration<br>Zeng, Yue; University of Hong Kong, Department of Social Work and Social Administration<br>Dai, Ruizhi; University of Hong Kong<br>Comas-Herrera, Adelina; London School of Economics and Political Science, Care Policy and Evaluation Centre; CPEC, London School of Economics and Political Sci<br>McDaid, David ; London School of Economics<br>Knapp, Martin; London School of Economics, Personal Social Services Research Unit<br>Wong, Gloria; Hong Kong University,
**Primary Subject Heading**:	Mental health
Secondary Subject Heading:	Health policy, Public health
Keywords:	Dementia < NEUROLOGY, Old age psychiatry < PSYCHIATRY, PUBLIC HEALTH, Health policy < HEALTH SERVICES ADMINISTRATION & MANAGEMENT

For peer review only - http://bmjopen.bmj.com/site/about/guidelines.xhtml

I, the Submitting Author has the right to grant and does grant on behalf of all authors of the Work (as defined in the below author licence), an exclusive licence and/or a non-exclusive licence for contributions from authors who are: i) UK Crown employees; ii) where BMJ has agreed a CC-BY licence shall apply, and/or iii) in accordance with the terms applicable for US Federal Government officers or employees acting as part of their official duties; on a worldwide, perpetual, irrevocable, royalty-free basis to BMJ Publishing Group Ltd ("BMJ") its licensees and where the relevant Journal is co-owned by BMJ to the co-owners of the Journal, to publish the Work in this journal and any other BMJ products and to exploit all rights, as set out in our licence.

The Submitting Author accepts and understands that any supply made under these terms is made by BMJ to the Submitting Author unless you are acting as an employee on behalf of your employer or a postgraduate student of an affiliated institution which is paying any applicable article publishing charge ("APC") for Open Access articles. Where the Submitting Author wishes to make the Work available on an Open Access basis (and intends to pay the relevant APC), the terms of reuse of such Open Access shall be governed by a Creative Commons licence – details of these licences and which Creative Commons licence will apply to this Work are set out in our licence referred to above.

Other than as permitted in any relevant BMJ Author's Self Archiving Policies, I confirm this Work has not been accepted for publication elsewhere, is not being considered for publication elsewhere and does not duplicate material already published. I confirm all authors consent to publication of this Work and authorise the granting of this licence.

For peer review only

Title page

Effectiveness of interventions for people living with dementia and their carers in Chinese communities: protocol for a systematic review and meta-analysis of randomised controlled trials

Cheng Shi[1,2], Shuangzhou Chen[1], Maximilian Salcher-Konrad[3], Jacky CP Choy[1], Hao Luo,[1,4] Dara KY Leung[1], Xinxin Cai[1], Yue Zeng[1], Ruizhi Dai[5], Adelina Comas-Herrera[3], David McDaid[3], Martin Knapp[3], Gloria HY Wong[1]

Correspondence to Gloria Hoi Yan Wong, 524 The Jockey Club Tower, Centennial Campus, The University of Hong Kong, Pokfulam, Hong Kong. Email: ghywong@hku.hk

Author affiliations

[1]Department of Social Work and Social Administration, The University of Hong Kong, Hong Kong

[2]Center for Social Welfare Studies, Beijing Normal University, Beijing, China

[3]Care Policy and Evaluation Centre, London School of Economics and Political Science, London, UK

[4]Department of Computer Science, The University of Hong Kong, Hong Kong

[5]Faculty of Social Sciences, The University of Hong Kong, Hong Kong

Authors contact

Cheng Shi (cs2019@hku.hk), Shuangzhou Chen (chenshuangzhou@hotmail.com), Maximilian Salcher-Konrad (m.salcher@lse.ac.uk), Jacky CP Choy (cpchoy@hku.hk), Hao Luo (haoluo@hku.hk), Dara KY Leung (daralky@hku.hk), Xinxin Cai (caixx@connect.hku.hk), Yue Zeng (yuezeng@connect.hku.hk), Ruizhi Dai (rdai@hku.hk), Adelina Comas-Herrera

For peer review only - http://bmjopen.bmj.com/site/about/guidelines.xhtml

(a.comas@lse.ac.uk), David McDaid (d.mcdaid@lse.ac.uk), Martin Knapp (m.knapp@lse.ac.uk), Gloria HY Wong (ghywong@hku.hk)

Author Contributions GHYW, CS, SC, JCPC, ACH, and MK defined the scope of the review and review question. GHYW, CS, SC, and MSK developed inclusion/exclusion criteria. HL designed the method for the Chinese bibliographical database search. GHYW, CS, SC, MSK, and DMD consulted on the bibliographical databases to be searched and search terms used. GHYW, CS, MSK, SC, JCPC, and DKYL provided information on methods of data synthesis. CS drafted the protocol. CS, SC, MSK, JCPC, HL, DKYL, XC, YZ, RD, ACH, DMD, MK, and GHYW contributed to the study design and reviewed the draft protocol. GHYW is the guarantor of the manuscript.

Funding This work is conducted as part of the "Tools to Inform Policy: Chinese Communities Actions in Response to Dementia" (TIP-CARD) project, supported by the Hong Kong Research Impact Fund of the Research Grants Council (Project Reference No.: R7017-18). MSK, DMD, and ACH's contributions were supported by the UK Research and Innovation's Global Challenges Research Fund (ES/P010938/1) as part of the Strengthening Responses to Dementia in Developing Countries (STRiDE) project. The funders were not involved in the development of this protocol.

Competing interests None declared

Patient consent for publication Not required.

Provenance and peer review Not commissioned; externally peer reviewed.

For peer review only - http://bmjopen.bmj.com/site/about/guidelines.xhtml

Word Count: 3999/4,000

For peer review only

For peer review only - http://bmjopen.bmj.com/site/about/guidelines.xhtml

Effectiveness of interventions for people living with dementia and their carers in Chinese communities: protocol for a systematic review and meta-analysis of randomised controlled trials

ABSTRACT

Introduction: As the largest and most rapidly ageing population, Chinese people are now the major driver of the continued growth in dementia prevalence globally. The need for evidence-based interventions in Chinese communities is urgent. Although a wide range of pharmacological and non-pharmacological interventions for dementia have been trialled in Chinese populations, the evidence has not been systemically synthesised. This systematic review and meta-analysis aim to map out the interventions for people living with dementia and their carers in Chinese communities worldwide and compare the effectiveness of these interventions.

Methods and analysis: This protocol followed the Preferred Reporting Items for Systematic Review and Meta-Analysis Protocols checklist. We will search Chinese (China National Knowledge Infrastructure, WanFang DATA) and English bibliographical databases (MEDLINE, EMBASE, PsycINFO, CINAHL Plus, Global Health, WHO Global Index Medicus, Virtual Health Library, Cochrane CENTRAL, Social Care Online, BASE, MODEM Toolkit, Cochrane Database of Systematic Reviews), complemented by hand searching reference lists. We will include studies evaluating the effectiveness of interventions for dementia or mild cognitive impairment in Chinese populations, using a randomised control trial design, and published between January 2008 and June 2020. We will use a standardised form to extract data and version 2 of the Cochrane risk-of-bias tool for randomized trials to assess the risk of bias of the included studies. Collected data will be fully interpreted with narrative synthesis and analysed using pairwise and network meta-

For peer review only - http://bmjopen.bmj.com/site/about/guidelines.xhtml

analyses to pool intervention effects where sufficient information is available. We will perform subgroup analysis and meta-regression to explore potential reasons for heterogeneity.

Ethics and dissemination: No formal ethics approval is required for this protocol. The findings will facilitate the development of studies on interventions for dementia and timely inform dementia policymaking and practice. Planned dissemination channels include peer-reviewed publications, conference presentations, public events, and websites.

PROSPERO registration number: CRD42019134135

For peer review only

Strengths and limitations of this study

- This systematic review and meta-analysis will be the first review of randomised control trials (RCTs) on the effectiveness of both pharmacological and non-pharmacological interventions for people living with dementia and their carers in Chinese communities worldwide.
- We will use a comprehensive search strategy of publications in both Chinese bibliographical databases (China National Knowledge Infrastructure, WanFang DATA) and English bibliographical databases (MEDLINE, EMBASE, PsycINFO, CINAHL Plus, Global Health, WHO Global Index Medicus, Virtual Health Library, Cochrane CENTRAL, Social Care Online, BASE, MODEM Toolkit, Cochrane Database of Systematic Reviews)
- We will narratively synthesise the collected data to map out the dementia-related interventions studied in Chinese communities, and conduct pairwise and network meta-analyses to compare the effectiveness of interventions.
- This review will be limited by the number and quality of RCTs conducted in Chinese communities.

For peer review only - http://bmjopen.bmj.com/site/about/guidelines.xhtml

INTRODUCTION

Around 50 million people currently live with dementia worldwide, of whom 20 percent are Chinese populations.[1] Chinese populations refer to people of Chinese ethnicity or national heritage, regardless of their nationality or region of residence. As the largest and most rapidly ageing population, the Chinese are now the major driver in the continued growth of global dementia prevalence.[2] Due to the physical and emotional challenges involved in caring, dementia not only affects people living with the condition, but also their families, formal carers, and other supporters.[3] With a culture emphasising filial piety, coupled with insufficient care services, family care is often the main supporting resource for people living with dementia (PLwD) in Chinese communities worldwide. Dementia has been recognised as one of the most burdensome diseases among Chinese populations.[4]

There is currently no cure for dementia, although symptoms can be managed with effective intervention and good care.[3] China recently launched its national dementia strategy, one of whose main tasks is to improve the wellbeing of PLwD by increasing service provision.[5] Taiwan updated its dementia policy in 2017, promoting dementia research, innovation, and development as one of its seven strategies.[6] In Macau's 10-year Plan of Action on Dementia Services published in 2016, strengthening community services and caregiver support comprises one of its five strategies.[7] In Hong Kong, a government services review and programme plan published in 2017[8,9] highlighted the need to strengthen services for PLwD and recommended a seven-stage model for dementia service following the World Health Organization and Alzheimer's Disease International's framework.[10] The need for evidence-based interventions and care services in Chinese populations is urgent.

For peer review only - http://bmjopen.bmj.com/site/about/guidelines.xhtml

Studies on dementia interventions appear to be scarce in Asian populations.[2] Most evidence on drug treatment and non-pharmacological interventions has been generated in Western countries, with questionable relevance for Chinese populations. For example, Cognitive Stimulation Therapy (CST) used alone or in combination with medication was shown to be effective and even cost-effective in improving cognition and quality of life, [11-14] leading to a recommendation for routine use by England's National Institute for Health and Care Excellence (NICE),[15] and by Alzheimer's Disease International.[16] In contrast, preliminary findings from a study applying CST with Hong Kong Chinese suggest that a larger number of participants needed to be treated to achieve clinically significant improvement in cognition.[17] Such discrepancies in an intervention's effect, possibly due to cultural differences, highlight the importance of generating evidence on the effectiveness of dementia-related interventions relevant to local populations.

There is now increasing evidence on a wide range of interventions for dementia undertaken in Chinese populations. A few reviews have been published, focusing on specific interventions and subtypes of dementia, such as the efficacy of donepezil in Chinese with Alzheimer's disease,[18] Chinese herbal medicine as adjunctive therapy for vascular dementia,[19] and traditional Chinese mind-body exercise (baduanjin) in older adults with mild cognitive impairment (MCI).[20] Growing evidence also suggests that the therapeutic response to dementia intervention (e.g., donepezil) might differ between Chinese and Western populations due to pharmacogenetic factors,[18,21] thus emphasising the need for more accurate evaluations of interventions tailored to Chinese populations.[22]

Some existing and ongoing studies aim to synthesise evidence for dementia intervention and care, including the Modelling Outcome and Cost Impacts of Interventions for Dementia (MODEM) project[23] with a dementia evidence toolkit (https://www.modem-dementia.org.uk/)

For peer review only - http://bmjopen.bmj.com/site/about/guidelines.xhtml

covering dementia interventions in English literature, and the Strengthening Responses to Dementia in Developing Countries (STRiDE) project (https://stride-dementia.org/) with an ongoing systematic review and meta-analysis on the evidence in seven low- and middle-income countries.[24] There is no comprehensive evidence synthesis on the effectiveness of dementia or dementia-related interventions that cover different types of dementia (e.g. Alzheimer's disease, vascular dementia, frontotemporal dementia, Lewy body disease and mixed dementia) and interventions (e.g. pharmacological treatment, psychosocial intervention, and traditional Chinese medicine) conducted in Chinese populations. Existing systematic reviews have focused mainly on the English language literature, where evidence from high-income areas such as Hong Kong and Taiwan can be found. Although Chinese academic databases have been recognised as a valuable resource for dementia-related studies, they have not been fully explored.[25-27]

To our knowledge, this will be the first systematic review and meta-analysis to comprehensively synthesise and assess the evidence on the effectiveness of interventions for PLwD and their carers among Chinese populations in Chinese and English bibliographical databases. We aim to 1) map out interventions for dementia studied in Chinese communities, and 2) compare the effectiveness of those interventions for achieving desired outcomes. This study will contribute to shape understanding of existing evidence on effectiveness of dementia-related interventions, improve quality of life of PLwD and their carers, and provide valuable information for practice, policymaking and further research. As part of a research project, Tools to Inform Policy: Chinese Communities Actions in Response to Dementia (TIP-CARD; www.tip-card.hku.hk/), this study also aligns with the above-mentioned dementia evidence synthesis effort by the STRiDE project.[24]

For peer review only - http://bmjopen.bmj.com/site/about/guidelines.xhtml

METHODS AND ANALYSIS

Protocol and registration

This protocol for this systematic review and meta-analysis followed the Preferred Reporting Items for Systematic Review and Meta-Analysis Protocols checklist.[28] This study has been registered on PROSPERO (www.crd.york.ac.uk/prospero; registration number: CRD42019134135).

Eligibility criteria

Population

We will include studies conducted among adults (aged 18 years and over) living with dementia or MCI and their carers in Chinese populations. We will include relevant studies conducted in any type of care settings, such as home, community, residential homes, clinics, hospitals, and other care settings. Participants characteristics such as gender, education, and age at diagnosis will not be used for excluding studies.

We will include studies covering people living with any type and stage of dementia. Dementia, as a major neurocognitive disorder, describes a group of symptoms of cognitive decline, including, but not limited to, Alzheimer's disease, vascular dementia, frontotemporal dementia, Lewy body disease, and mixed dementia. Studies conducted among people living with MCI, mild neurocognitive disorder, vascular cognitive impairment, and no dementia (VCIND) will be eligible for inclusion due to the higher risk of developing dementia in later years.[29] We will also include studies conducted with people with diseases co-occurring with dementia and studies not specifying the type of dementia. However, we will exclude studies without explicating the diagnostic criteria for dementia or MCI when recruiting participants.

For peer review only - http://bmjopen.bmj.com/site/about/guidelines.xhtml

Our definition of dementia carer refers to persons involved in care provision and management and will not depend on whether or not the carer is paid, lives with the person they care for, or provide direct or indict care. Therefore, dementia carers includes health and social care professionals, care managers, care workers, administrative staff of care facilitators, family carers, other unpaid carers, and family members assisting with care decisions. We will focus on studies conducted among people of Chinese ethnicity or national heritage regardless of their nationality or location of residence. Studies without explicating the proportion of Chinese participants over 50% or studies without a specific subgroup analysis for Chinese participants will be excluded.

Intervention

Based on the effectiveness perspective,[24,30] any type of interventions for improving desired outcomes will be eligible. We will include studies on pharmacological treatment, non-pharmacological intervention (e.g., cognitive intervention, technological intervention, training, and exercise), or multicomponent interventions. We will exclude studies: 1) where no clear intervention was described, 2) on primary prevention of dementia, and 3) on non-interventional studies.

Comparison

Any type of comparisons will be eligible for inclusion, such as active comparators, treatment as usual, placebo, and no treatment.

Outcomes

For peer review only - http://bmjopen.bmj.com/site/about/guidelines.xhtml

Any type of outcomes of dementia-related intervention will be eligible for inclusion from the perspective of effectiveness, which may affect individuals, families, the dementia care workforce, wider society, and social or health care systems. Dementia often triggers complex problems in many domains.[22] According to the MODEM dementia evidence toolkit (https://www.modem-dementia.org.uk/), outcomes measured in existing studies may include 1) cognition, behavioural and psychological symptoms, functional status, physical health and quality of life of PLwD; 2) carer burden, the carer's mental health, quality of life and other carer outcomes (e.g., financial burdens); 3) service use, cost reduction (including hospital use reduction and care home admission delay) and service satisfaction; 4) risk reduction (of dementia and co-morbidities) and prevention or management of comorbidities. To capture the diversity of interventions trialled in Chinese communities, we will accept all outcome measures that reflect intervention effectiveness.

Study design

To identify potential causal relationships, we will only include studies using randomised controlled trial (RCT) or cluster RCT designs. To control study quality, we will only include RCTs with a low risk of bias in the process of evidence generation. According to version 2 of the Cochrane risk-of-bias tool for randomized trials (RoB 2), [31] methods used for generating random allocation sequence indicating low risk of bias include computer-generated random numbers, a random number table, coin tossing, shuffling cards or envelopes, throwing dice, or drawing lots. Studies will be excluded that use no random element or provide no information on the generation process of the random allocation sequence.

To minimise small-study effects,[32,33] we will exclude studies with a sample size of less than 50 in either the intervention group or comparison group(s) for the eligible population. For

For peer review only - http://bmjopen.bmj.com/site/about/guidelines.xhtml

studies conducted with a population of mixed ethnicity, the sample size of each study arm for Chinses sub-group analysis should be greater than 50 participants. For studies in which more than 50% of participants are Chinese and all participants are randomly grouped, the sample size of each study arm is expected to be greater than 50 participants regardless of ethnicity.

Publication type

We will include the primary publications of intervention studies and grey literature provided they have evaluated the effectiveness of dementia-related interventions in Chinese populations. Relevant systematic reviews or scoping reviews will be included in the first step screening, and then will be used to complement the primary publications by a manual search of the reference lists. Conference abstracts will be included only if they contain sufficient information to assess eligibility for inclusion.

Publication period

Studies published between January 2008 and June 2020.

Language

Studies will be limited to English and Chinese language publications.

Information sources

We will search two major Chinese bibliographical databases (China National Knowledge Infrastructure and WanFang DATA) and English bibliographical databases (MEDLINE,

For peer review only - http://bmjopen.bmj.com/site/about/guidelines.xhtml

EMBASE, PsycINFO, CINAHL Plus, Global Health, WHO Global Index Medicus, Virtual Health Library, Cochrane CENTRAL, Social Care Online, BASE, MODEM Toolkit, Cochrane Database of Systematic Reviews). Hand searching of reference lists among reviewed studies will complement the database searches.

Search strategy

We will adapt an established search strategy protocol[24] used to search for English language literature. Corresponding Chinese search terms will be translated and adapted by three bilingual researchers (GHYW, SC, and CS) experienced in dementia/ageing research with a training background in psychology, psychiatry, translation, social work, and social policy from Hong Kong and mainland China. Search terms in English and Chinese are listed in Table 1. In studies published in English, the search terms related to Chinese populations include "China", "Chinese", "Sino", "Hong Kong", "Taiwan", "Taiwanese", "Macau", and "Asian".

For studies published in English, we will first extract eligible study records identified from an ongoing systematic review,[24] which used the same search strategy and search terms for dementia intervention and covered studies published between 2008 and 2018. Then we will search these terms for Chinese populations in the title, abstract, and keywords. Second, we will repeat the English bibliographical database search mentioned above to identify studies published between January 2019 and June 2020.

For studies published in Chinese, we will use Python,[34] a programming language, to facilitate Chinese bibliographical database searching by using dementia-related search terms (search items #1-4 in Table 1). This is because of the technical challenge posed by limitations on the number of search terms and exported records per time in the two Chinese bibliographical

For peer review only - http://bmjopen.bmj.com/site/about/guidelines.xhtml

databases. The search results for dementia-related study records will be exported in a Microsoft Excel spreadsheet. Then we will search the intervention-related terms (search items #5-53) in the title and abstract.

Study records

Data management

To deal with a potentially large number of search results and various data sources in two languages, we will manage references using two web-based software packages during the review and extraction process: 1) Rayyan (https://rayyan.qcri.org/), a web and mobile app that can facilitate the initial screening of abstracts and titles using a semi-automated process,[35] and 2) Covidence (https://www.covidence.org/), an internet-based software platform for managing systematic reviews, including study selection, risk of bias assessment, and data extraction.

Duplicate publications will be checked based on title, author, journal, and year using the Microsoft Excel, and the "Find duplicates" function in Rayyan and Covidence. Multiple publications from the same study will be identified based on the key information (e.g., authors' names, study design, intervention, and outcomes) from the full-texts, or by contacting authors for clarification if needed. Once confirmed, included multiple publications will be linked on Covidence.

Study selection

Study selection will be a two-step process, with detailed explanations for inclusion and exclusion criteria in each step. First, two researchers will independently screen the title and abstract and

For peer review only - http://bmjopen.bmj.com/site/about/guidelines.xhtml

determine the study's inclusion or exclusion on Rayyan. A justification (criterion) will be required for any exclusion decision. Studies with insufficient information in the title and abstract to enable a decision to be made will be included at this stage. The Rayyan machine learning-based classifier[35] will be considered to facilitate the title and abstract screening, given the potentially work overload. Using a certain number of manually screened studies as a training dataset, Rayyan will generate a relevance rating for each study, ranging from 0.5 (lowest) to 5 (highest).[35] We may use a low relevance score (e.g., below 1.5) as a threshold to guide study exclusion.

Second, studies included following title and abstract screening will be uploaded to Covidence for full-text review by two independent reviewers, who will provide a justification for each excluded study. Review studies will be excluded at this stage, although their reference lists will be used to complement the database search results.

All disagreements in each step will be resolved through discussion between the two reviewers. If consensus is unreachable, a third reviewer will be consulted for a final decision.

Reviewers for title and abstract screening and full-text review will be able to read and understand inclusion/exclusion criteria for publications in both English and simplified Chinese.

Upon completion of the selection process, we will generate a Preferred Reporting Items for Systematic Reviews and Meta-Analyses flowchart[36] to illustrate the inclusion and exclusion of studies at each stage in study selection.

Data collection process

We will use a standardised form based on the template available from Covidence for data extraction that will be pilot-tested using included studies. To ensure data consistency across reviewers, we will organise exercises and group discussions for reviewer training. Due to the

For peer review only - http://bmjopen.bmj.com/site/about/guidelines.xhtml

anticipated large number of potentially eligible studies, the data extraction form will be completed by one reviewer and verified by the second reviewer. We will keep all records of corrections or amendments to the data extraction. In studies that do not report the required information, we will contact the authors to request information.

We will prepare the data extraction form in English. For studies in Chinese, our bilingual reviewers will complete data extraction using the original expression in Chinese full-texts except for the outcome name and brief introduction of the intervention, which will be recorded in English based on the English abstract if available or personal translation. The final extracted evidence from both the English and Chinese studies will be verified by one bilingual researcher (CS) to ensure consistent translation.

Data items

We will extract information on items listed in Box 1 from the included studies.

Outcomes and prioritisation

In line with our research aims, we will first record all types of outcome and outcome measures stated in the included studies to map out the dementia-related interventions conducted in Chinese communities. Due to the anticipated number of Chinese studies from an ongoing review,[24] we will prioritise primary and secondary outcomes as outcomes of interest when extracting outcome results from included studies. We will evaluate and compare the effectiveness of dementia-related interventions for outcomes of interest to ensure feasibility. However, the outcome of interest may need to change subject to the characteristics and the number of included studies. Details of proposed outcomes of interest are described below.

For peer review only - http://bmjopen.bmj.com/site/about/guidelines.xhtml

As dementia is a condition affecting cognition by definition, our primary outcome of interest in studies conducted among PLwD will be changes in cognition. Common assessments for measuring cognitive impairment level or performance include the Mini–Mental State Examination (MMSE), the Montreal Cognitive Assessment (MoCA), and the Alzheimer's Disease Assessment Scale-Cognitive Subscale (ADAS-Cog). Given the effects of dementia on the ability to organise activities,[22] we will also focus on changes in functional ability following treatment. For example, the Disability Assessment for Dementia (DAD) is designed for evaluating functional ability to complete activities of daily living (ADL) and instrumental activities of daily living (IADLs) among people living with dementia.

Caring for a person living with dementia can be very stressful, which may lead to a higher level of depression or health issues.[37] For studies conducted among carers of PLwD, we will focus on changes in quality of life and carer burden,[38,39] as measured by tools such as the EUROHIS-QOL 8-item index and the Zarit Burden Interview (ZBI), respectively.

If outcome of interest or its measure is not reported in included studies, we will extract the outcome results that are reported as the primary outcome of the original study.

We will extract results of primary and secondary outcomes measured at each time point reported in included studies. Nevertheless, we will afford preference to the endpoint of the study in the main data synthesis. Results at multiple time points will be used for subgroup analysis and meta-regression to explore the short-term and long-term effects of outcomes.

Risk of bias in individual studies

We will use the Cochrane Collaboration’s recently updated risk-of-bias tool (RoB 2)[31] to assess the quality of included studies in Covidence. Two reviewers will make independent judgements

For peer review only - http://bmjopen.bmj.com/site/about/guidelines.xhtml

based on the criteria for judging the risk of bias (RoB). Disagreement will be resolved by discussion and arbitrated by a third reviewer if consensus is unreachable. For studies that do not provide sufficient information in full-texts for RoB assessment, we will search for the study's protocol, trial registry information, or other relevant materials to facilitate the judgement. The absence of a pre-specified analysis plan may raise some concerns in the domain for bias in selection of the reported result.

Data synthesis

Evidence on dementia-related interventions in PLwDs and carers will be analysed separately. Studies of family-based or dyadic interventions involving both PLwDs and carers will be categorised according to the subject of each outcome.

Narrative synthesis

To map dementia-related interventions conducted in Chinese communities, we will undertake a narrative synthesis to fully interpret the extracted evidence from all included studies. We will first describe and summarise disease characteristics, features of the intervention, number of participants, participant characteristics, outcomes, outcome measures, and indication of risk of bias assessment in a tabular form. In line with the Guidance on the Conduct of Narrative Synthesis in Systematic Review,[40] we will then explore the relationship among types of interventions (or details of pharmacological, non-pharmacological and multicomponent interventions), outcomes, and outcome measures conducted in Chinese populations. Idea webbing will be used to visually describe conceptual linkages through examination of extracted data if feasible. The key questions here are what (types of) interventions have been conducted in Chinese communities, what specific

For peer review only - http://bmjopen.bmj.com/site/about/guidelines.xhtml

For peer review only

outcomes those interventions target and what measures are used for those outcomes. We expect to identify research gaps in this field for future studies and practice.

Meta-analyses

To compare the effectiveness of interventions for outcomes of interest (described in the 'Outcome and prioritisation' section), we will conduct quantitative synthesis of treatment effects through meta-analyses where sufficient information is available. For a specific outcome, we will perform a series of pairwise meta-analyses for all direct comparisons (e.g., one comparison between an intervention group and a control or another intervention group).[41] Due to the underlying difference between studies in terms of participants, intervention details and care settings, a random-effects pooling model will be conducted by default for an overall summary estimate by weighting studies using a combination of within- and between-study variance. When the included studies use different instruments to evaluate the same outcome (e.g., MMSE, MoCA, and ADAS-Cog for measuring cognition), we will use standardised mean difference (the absolute mean difference between the intervention group and control group divided by the standard deviation in the control group) for continuous outcomes and relative risks for dichotomous outcomes to compute the effect size for each study.

To compare the effectiveness for multiple interventions, we will use network meta-analysis to combine direct and indirect evidence for relevant treatment effects.[42] In network meta-analyses, different comparisons among two or more of the treatments can be included in one analysis. We will generate network geometry to visualise and assess the treatment networks, and estimate and combine comparative effects from direct and indirect evidence. In examining the transitivity hypothesis of network meta-analysis, we will use 'loop-specific approaches' to detect the inconsistency of a network of interventions, including local inconsistency test to evaluate the

For peer review only - http://bmjopen.bmj.com/site/about/guidelines.xhtml

loop inconsistency in regions of network separately[43] and global inconsistency test to evaluate the incoherence in the overall network.[44]

Sensitivity analyses will be conducted to explore the robustness of the meta-analysis results by varying the analytic data or methods, including analysing studies only with a low risk of bias and trails using a placebo as a comparator.[45]

Dealing with missing data

When there are missing data, we will attempt to obtain these by contacting the study author(s). If unsuccessful, we will consider using imputation methods to impute the missing value[46] or exclude studies with missing data from the quantitative analysis. We will use sensitivity analysis to evaluate the potential influence on the overall treatment effects of included studies that use per-protocol analysis or suggest the result was biased by missing outcome data (i.e., high risk of bias) based on the RoB 2 assessment tool.[31]

Subgroup analysis and meta-regression

We will calculate Cochran's Q statistic and the I^2 statistic to estimate the heterogeneity of the included studies.[47] If statistical heterogeneity is observed, we will conduct subgroup analysis and meta-regression to explore the potential reasons for the differences. Potential candidate covariates for subgroup analysis and meta-regression include intervention characteristics (e.g., types of intervention, intervention dosage, and duration), participant characteristics (e.g., age, gender, education, severity of dementia, and type of dementia), care settings, follow-up period

For peer review only - http://bmjopen.bmj.com/site/about/guidelines.xhtml

(e.g., at three, six, and 12 months) and locations (e.g., mainland China, Hong Kong, Taiwan, Macau, and other Chinese communities worldwide).

Meta-bias(es)

For each meta-analysis, we will use a funnel plot asymmetry assessment to detect meta-biases. Statistical tests for funnel plot asymmetry will be performed when at lease10 studies included in the meta-analysis.[48] Contour lines indicating various statistical significance will be used to aid visual interpretation of funnel plots. If funnel plot asymmetry is observed, we will also consider other possible reasons apart from non-reporting bias such as poor methodological quality and true heterogeneity of the included studies.[49]

Confidence in cumulative estimate

We will use the Grading of Recommendations Assessment, Development and Evaluation (GRADE) approach[50] to assess the quality of evidence. The domains of the assessment include risk of bias, inconsistency, indirectness of evidence, imprecision, and publication bias.

Patient and public involvement

Neither patients nor the public will be involved in the design or development of this review protocol. However, stakeholders, including PLwD, family members, care staff, healthcare professionals, and policymakers, will be engaged in the dissemination plan as described below.

ETHICS AND DISSEMINATION

For peer review only - http://bmjopen.bmj.com/site/about/guidelines.xhtml

This protocol for a systematic review and meta-analysis describes the methods to identify and synthesize published evidence on the effectiveness of interventions for PLwD and their carers in Chinese communities. No formal ethics approval is required for this protocol. The findings from this review will facilitate the development of studies on interventions for dementia and provide timely information for dementia policymaking and practice. We will target both professionals and non-specialist audiences in disseminating the outcomes of the review through print and events, including peer-reviewed publications, conference presentations, public events, and publicly accessible websites.

REFERENCES

1. Patterson C. World Alzheimer Report 2018: The State of the Art of Dementia Research: New Frontiers. London: Alzheimer's Disease International, 2018.

2. Prince M, Wimo A, Guerchet M, et al. World Alzheimer Report 2015: The Global Impact of Dementia. London: Alzheimer's Disease International, 2015.

3. Livingston G, Sommerlad A, Orgeta V, et al. Dementia prevention, intervention, and care. *Lancet* 2017;390(10113):2673-734. doi: 10.1016/S0140-6736(17)31363-6

4. Lou VWQ, Chi I. Is China Ready For Dementia. *Alzheimers and Neurodegenerative Diseases* 2020:6(1/2): 041. doi: 10.24966/AND-9608/100041

5. National Health Commission. Work Plan on Exploring Special Services for Senile Dementia Prevention and Treatment (Tansuo laonian chidai fangzhi tese fuwu gongzuo fang'an), 2020.

6. Ministry of Health and Welfare, Republic of China (Taiwan),. Taiwan Dementia Policy: A Framework for Prevention and Care 2.0, 2017.

For peer review only - http://bmjopen.bmj.com/site/about/guidelines.xhtml

For peer review only

7. Government of Macao SAR. 10-Year Plan of Action on Services for Older Persons 2016-2025. Macao Special Administrative Region Government, 2016.

8. Food and Health Bureau. Mental Health Review Report. Hong Kong: Food and Health Bureau, Hong Kong Special Administrative Region Government, 2017.

9. Working Group on Elderly Services Programme Plan, Elderly Commission of Government of Hong Kong SAR,. Elderly Services Programme Plan, 2017.

10. World Health Organization. Dementia: A Public Health Priority. Geneva: World Health Organization, 2012.

11. Knapp M, Thorgrimsen L, Patel A, et al. Cognitive stimulation therapy for people with dementia: cost-effectiveness analysis. *Brit J Psychiat* 2006;188:574-80. doi: 10.1192/bjp.bp.105.010561

12. Woods B, Aguirre E, Spector AE, et al. Cognitive stimulation to improve cognitive functioning in people with dementia. *Cochrane Db Syst Rev* 2012(2) doi: 10.1002/14651858.CD005562.pub2

13. Huntley JD, Gould RL, Liu K, et al. Do cognitive interventions improve general cognition in dementia? A meta-analysis and meta-regression. *Bmj Open* 2015;5(4) doi: 10.1136/bmjopen-2014-005247

14. D'Amico F, Rehill A, Knapp M, et al. Maintenance Cognitive Stimulation Therapy: An Economic Evaluation Within a Randomized Controlled Trial. *J Am Med Dir Assoc* 2015;16(1):63-70. doi: 10.1016/j.jamda.2014.10.020

15. National Institute for Health and Care Excellence. Dementia: Assessment, Management and Support for People Living with Dementia and their Carers. London: NICE, 2018.

For peer review only - http://bmjopen.bmj.com/site/about/guidelines.xhtml

16. Alzheimer's Disease International. World Alzheimer Report 2011: The Benefits of Early Diagnosis and Intervention: London: Alzheimer's Disease International, 2011.

17. Wong GHY, Yek OPL, Zhang AY, et al. Cultural adaptation of cognitive stimulation therapy (CST) for Chinese people with dementia: multicentre pilot study. *Int J Geriatr Psych* 2018;33(6):841-48. doi: 10.1002/gps.4663

18. Zhang N, Gordon ML. Clinical efficacy and safety of donepezil in the treatment of Alzheimer's disease in Chinese patients. *Clin Interv Aging* 2018;13:1963-70. doi: 10.2147/CIA.S159920

19. Zeng LF, Zou YP, Kong LS, et al. Can Chinese herbal medicine adjunctive therapy improve outcomes of senile vascular dementia? Systematic review with meta-analysis of clinical trials. *Phytother Res* 2015;29(12):1843-57. doi: 10.1002/ptr.5481

20. Xia R, Qiu PT, Lin HY, et al. The effect of traditional Chinese mind-body exercise (Baduanjin) and brisk walking on the dorsal attention network in older adults with mild cognitive impairment. *Front Psychol* 2019;10:2075 doi: ARTN 207510.3389/fpsyg.2019.02075

21. Liu HC, Hong CJ, Wang SJ, et al. ApoE genotype in relation to AD and cholesterol - A study of 2,326 Chinese adults. *Neurology* 1999;53(5):962-66. doi: Doi 10.1212/Wnl.53.5.962

22. Livingston G, Huntley J, Sommerlad A, et al. Dementia prevention, intervention, and care: 2020 report of the Lancet Commission. *Lancet* 2020;396(10248):413-46. doi: 10.1016/S0140-6736(20)30367-6

23. Comas-Herrera A, Knapp M, Wittenberg R, et al. MODEM: A comprehensive approach to modelling outcome and costs impacts of interventions for dementia. Protocol paper. *BMC Health Serv Res* 2017;17(1):25. doi: 10.1186/s12913-016-1945-x

For peer review only - http://bmjopen.bmj.com/site/about/guidelines.xhtml

24. Salcher-Konrad M, Naci H, McDaid D, et al. Effectiveness of interventions for dementia in low- and middle-income countries: protocol for a systematic review, pairwise and network meta-analysis. *BMJ Open* 2019;9(6):e027851. doi: 10.1136/bmjopen-2018-027851

25. Fung ICH. Chinese journals: a guide for epidemiologists. *Emerg Themes Epidemi* 2008;5:20 doi: 10.1186/1742-7622-5-20

26. Xia J, Wright J, Adams CE. Five large Chinese biomedical bibliographic databases: accessibility and coverage. *Health Info Libr J* 2008;25(1):55-61. doi: 10.1111/j.1471-1842.2007.00734.x

27. Chan KY, Wang W, Wu JJ, et al. Epidemiology of Alzheimer's disease and other forms of dementia in China, 1990-2010: a systematic review and analysis. *Lancet* 2013;381(9882):2016-23. doi: 10.1016/S0140-6736(13)60221-4

28. Shamseer L, Moher D, Clarke M, et al. Preferred reporting items for systematic review and meta-analysis protocols (PRISMA-P) 2015: elaboration and explanation. *BMJ* 2015;350:g7647. doi: 10.1136/bmj.g7647 [published Online First: 2015/01/04]

29. Mitchell AJ, Shiri-Feshki M. Rate of progression of mild cognitive impairment to dementia–meta-analysis of 41 robust inception cohort studies. *Acta Psychiatrica Scandinavica* 2009;119(4):252-65.

30. Reeves BC, Deeks JJ, Higgins J. 13 Including non-randomized studies. *Cochrane handbook for systematic reviews of interventions* 2008;1:391.

31. Sterne JAC, Savovic J, Page MJ, et al. RoB 2: a revised tool for assessing risk of bias in randomised trials. *BMJ* 2019;366:l4898. doi: 10.1136/bmj.l4898 [published Online First: 2019/08/30]

For peer review only - http://bmjopen.bmj.com/site/about/guidelines.xhtml

32. Chen HY, Shen DT, Ji DZ, et al. Prevalence and burden of hepatitis D virus infection in the global population: a systematic review and meta-analysis. *Gut* 2019;68(3):512-21. doi: 10.1136/gutjnl-2018-316601 [published Online First: 2018/09/20]

33. Turner RM, Bird SM, Higgins JP. The impact of study size on meta-analyses: examination of underpowered studies in Cochrane reviews. *PLoS One* 2013;8(3):e59202. doi: 10.1371/journal.pone.0059202 [published Online First: 2013/04/02]

34. Python Software Foundation. Python language reference, version 3.6. 8. 2020

35. Ouzzani M, Hammady H, Fedorowicz Z, et al. Rayyan—a web and mobile app for systematic reviews. *Syst Rev* 2016;5(1):210. doi: 10.1186/s13643-016-0384-4

36. Moher D, Liberati A, Tetzlaff J, et al. Preferred Reporting Items for Systematic Reviews and Meta-Analyses: The PRISMA Statement. *PLOS Medicine* 2009;6(7):e1000097. doi: 10.1371/journal.pmed.1000097

37. Baumgarten M, Battista RN, Infanterivard C, et al. The psychological and physical health of family members caring for an elderly person with dementia. *J Clin Epidemiol* 1992;45(1):61-70. doi: Doi 10.1016/0895-4356(92)90189-T

38. Chien WT, Lee IYM. Randomized controlled trial of a dementia care programme for families of home-resided older people with dementia. *J Adv Nurs* 2011;67(4):774-87. doi: 10.1111/j.1365-2648.2010.05537.x

39. Lam LCW, Lee JSW, Chung JCC, et al. A randomized controlled trial to examine the effectiveness of case management model for community dwelling older persons with mild dementia in Hong Kong. *Int J Geriatr Psych* 2010;25(4):395-402. doi: 10.1002/gps.2352

40. Popay J, Roberts H, Sowden A, et al. Guidance on the conduct of narrative synthesis in systematic reviews. *A product from the ESRC Methods Programme* 2006;1:b92.

For peer review only

For peer review only - http://bmjopen.bmj.com/site/about/guidelines.xhtml

41. Lau J, Ioannidis JP, Schmid CH. Quantitative synthesis in systematic reviews. *Ann Intern Med* 1997;127(9):820-6. doi: 10.7326/0003-4819-127-9-199711010-00008 [published Online First: 1998/02/12]

42. Cuijpers P, Noma H, Karyotaki E, et al. Effectiveness and acceptability of cognitive behavior therapy delivery formats in adults with depression: a network meta-analysis. *JAMA Psychiatry* 2019;76(7):700-07. doi: 10.1001/jamapsychiatry.2019.0268

43. Chaimani A, Higgins JPT, Mavridis D, et al. Graphical Tools for Network Meta-Analysis in STATA. *Plos One* 2013;8(10) doi: 10.1371/journal.pone.0076654

44. Higgins JPT, Jackson D, Barrett JK, et al. Consistency and inconsistency in network meta-analysis: concepts and models for multi-arm studies. *Res Synth Methods* 2012;3(2):98-110. doi: 10.1002/jrsm.1044

45. Centre for Reviews and Dissemination. CRD's guidance for undertaking reviews in healthcare: CRD, University of York 2009.

46. Crameri A, von Wyl A, Koemeda M, et al. Sensitivity analysis in multiple imputation in effectiveness studies of psychotherapy. *Front Psychol* 2015;6 doi: 10.3389/fpsyg.2015.01042

47. Higgins JP, Thompson SG. Quantifying heterogeneity in a meta-analysis. *Stat Med* 2002;21(11):1539-58. doi: 10.1002/sim.1186 [published Online First: 2002/07/12]

48. Sterne JA, Sutton AJ, Ioannidis JP, et al. Recommendations for examining and interpreting funnel plot asymmetry in meta-analyses of randomised controlled trials. *Bmj* 2011;343

49. Egger M, Smith GD, Schneider M, et al. Bias in meta-analysis detected by a simple, graphical test. *Bmj* 1997;315(7109):629-34.

50. Schünemann H, Brożek J, Guyatt G, et al. Handbook for Grading the Quality of Evidence and the Strength of Recommendations using the GRADE Approach: Cochrane Collaboration;

For peer review only

For peer review only - http://bmjopen.bmj.com/site/about/guidelines.xhtml

2013 [Available from: https://gdt.gradepro.org/app/handbook/handbook.html accessed on 19 Oct 2020.

For peer review only

For peer review only - http://bmjopen.bmj.com/site/about/guidelines.xhtml

TABLES

Table 1 Search terms related to dementia and intervention in English and Simplified Chinese

Search #	Search terms in English	Search terms in Simplified Chinese
1	Dementia	痴呆 or 失智 or 认知症
2	Cognitive disorder	认知障碍 or 认知功能障碍 or 认知紊乱 or 认知功能紊乱
3	Alzheimer	茨海默 or 兹海默
4	((cognit* or memory or cerebr*) adj3 (impair* or los* or declin* or deteriorat* or degenerat*)).mp.	(认知 or 记忆 or 脑) (缺损 or 缺失 or 退*化 or 衰退 or 下降 or 损伤 or 恶化 or 损害 or 退行)
5	(Intervention* or therap* or treatment* or program* or manage* or prevent* or diagnos* or polic*).mp.	干预 or 介入 or 治疗 or 疗法 or 方案 or 处理 or 预防 or 诊断 or 措施 or 手段 or 政策 or 应用 or 支持 or 效果 or 疗效 or 观察 or 价值 or 临床 or 分析
6	Cognitive therapy	认知 (治疗 or 疗法)
7	Cognitive stimulation	认知 (刺激 or 促进)
8	Cognitive training	认知训练
9	Cognitive rehabilitation	认知 (复康 or 复健 or 康复)
10	Drug therapy or pharmacotherapy	*药*
11	Cholinesterase inhibitors	胆碱分解抑制剂 or 胆碱酵素抑制剂 or 胆碱酶抑制剂
12	Cholinesterase agent	胆碱分解剂 or 胆碱酵素剂 or 胆碱酶剂
13	(Sedative or tranquili* adj3 (agent* or drug*)).mp.	(镇静 or 镇定 or 安神 or 安定) (药 or 剂)
14	Antipsychotic or neuroleptic (agent* or drug*)	抗精神病 (药 or 剂)
15	exp Serotonin Reuptake Inhibitors or ssri	(血清素 or 5-羟色胺) (再摄取 or 再吸收 or 回收) 抑制剂
16	Benzodiazepines	苯二氮平 or 苯二氮卓
17	(memantine or donepezil or rivastigmine or galantamine or souvenaid or risperidone or haloperidol or olanzapine	(美金刚 or 美金胺) (多奈哌齐 or 多奈哌其) (卡巴拉汀 or 利斯的明) (加兰他敏 or 加兰他明 or 格兰他明) (智敏捷) (利培酮 or 利螺环酮) (氟哌啶醇 or 氟哌丁苯 or 氟哌醇 or 卤吡醇) (奥氮平) (喹硫平) (西酞普兰) (右美沙芬 or 右旋美沙酚 or 右旋美索芬 or 右甲吗喃) (卡馬西

For peer review only - http://bmjopen.bmj.com/site/about/guidelines.xhtml

	or quetiapine or citalopram or dextromethorphan or carbamazepine or mirtazapine or sertraline or moclobemide or trazodone or melatonin or ramelteon or methylphenidate).mp.	平 or 卡马平 or 卡巴氮平 or 卡巴马平) (米氮平) (舍曲林) (吗氯贝胺) (曲唑酮) (褪黑素 or 褪黑激素) (雷美替胺 or 拉米替隆) (哌甲酯 or 派醋甲酯 or 盐酸甲酯)
18	Movement Therapy	(运动 or 动作)
19	(Physical activit* or physical training).mp.	(运动 or 体育 or 体能) (活动 or 训练)
20	(social adj3 activit*).mp.	社交活动 or 社会活动
21	Psychotherapy	心理 (治疗 or 疗法)
22	(behavio?r* adj3 therap*).mp.	行为 (治疗 or 疗法)
23	Counseling	辅导 or 咨询
24	((Psychosocial or psycho social) adj3 (support or interven* or care)).ti,ab.	（社会心理 or 社交心理）(支援 or 治疗 or 干预 or 介入 or 照顾)
25	Alternative medicine	(替代 or 另类) (治疗 or 疗法 or 医学 or 医疗)
26	Chinese medicine	中医 or 中药
27	Acupuncture	针灸 or 针刺 or 电针
28	(herb* adj3 (tea or remedy or remedies or medicine*)).ti,ab.	草药 or 药草 or 药用植物 or 草本 or 茶疗
29	Gingko	银杏 or 白果
30	homeopathy	(顺势 or 同质 or 同种)
31	((music or art or aroma or light or photo or pet or pets) adj3 therap*).ti,ab.	(音乐 or 艺术 or 香薰 or 光照 or 光线 or 宠物 or 动物 or 舞蹈)
32	Massage	按摩 or 推拿
33	Mind Body Therapy	身心 or 心身 or 正念 or 冥想
34	Advance directives	预设医疗指示 or 预设指示 or 预前意愿 or 预先指示
35	(Advance? adj3 (care or medical or healthcare) adj3 plan*).mp.	(预设 or 预立) (护理计划 or 临终*计划 or 医疗决定)
36	(decision* adj3 (aid* or support)).mp.	决策援助 or 决策辅助 or 决策支持
37	Case Management	个案管理
38	(communicati* adj3 skill* adj3 training).mp.	沟通技巧 (训练 or 培训)

For peer review only

For peer review only - http://bmjopen.bmj.com/site/about/guidelines.xhtml

39	(dementia care adj3 map*).mp.	认知障碍症照顾图谱 or 老年痴呆症照顾图谱 or 失智症照顾测绘
40	((person* or patient*) adj3 cent* adj3 care).mp.	(以人为本 or 人本 or 以人为中心 or 病人为本) (照顾 or 照护 or 护理 or 治疗 or 医疗)
41	((caregiver or carer) adj3 educat*).mp.	(照顾者 or 家属 or 家庭 or 照护者 or 照料者) 教育
42	Support Groups	（支援 or 支持 or 互助）（小组 or 组）
43	Self-Help Techniques	自助法 or 自助*法 or 自助技巧 or 自救*
44	Social Support	社交支援/社交支持/互助组
45	Computer assisted diagnosis	电脑辅助诊断/计算机辅助诊断
46	Telemedicine	远程医疗 or 远距医疗 or 远距离医疗
47	Computer Assisted Therapy	电脑辅助治疗 or 计算机辅助治疗
48	Mobile Devices	移动设备 or 行动装置
49	((smart adj2 (phone* or device* or tablet*)) or smartphone*).mp.	智能 (手机 or 电话 or 平板电脑) or 智能* or 可穿戴*
50	cognitive aid	认知辅助/认知帮助
51	Reminder	提示 or 提醒
52	Robot	机器人
53	Animal Experiment	动物实验/动物试验

For peer review only

For peer review only - http://bmjopen.bmj.com/site/about/guidelines.xhtml

Box 1 Data to be extracted from included studies

General information
- Reviewers' name
- Date of data extraction
- Publication details and identification
- Sponsorship source
- Research site: places (city-level) where the trial was conducted
- Setting (e.g., hospital, care home, community)
- Study aim(s)
- Publication language: Chinese or English

Methods
- Study design
- List of all outcomes with instruments reported in study

Population
- Inclusion criteria
- Exclusion criteria
- Group differences
- Clinical features (e.g., types of dementia, severity and duration of dementia)
- Baseline characteristics of participants in each study arm or overall participants: demographics (e.g., age, gender), socio-economic status (e.g., education), clinical outcomes if any, number of participants

Intervention
- Description of the intervention(s) and comparator(s) (e.g., name, treatment dose, duration, components and how it was delivered).
- Intervention type (e.g., pharmaceutical intervention, traditional Chinese medicine, non-pharmacological treatment and multicomponent interventions).

Outcomes
- Outcome name including name of each outcome of interest and how it was measured (instruments used)
- Outcome type and reported format. The components of reporting effect measures are: (1) the effect measure itself (e.g., change from baseline), (2) a measure of its variance (e.g., the standard deviation, or the 95% CI), (3) the number of participants in the study arm (N).
- Scale and direction of effect

Results
- Results of outcomes reported in the original study at each time point.

Risk of bias information
- Judgements based on the criteria of the RoB 2.

For peer review only - http://bmjopen.bmj.com/site/about/guidelines.xhtml

Reporting checklist for protocol of a systematic review.

Based on the PRISMA-P guidelines.

Instructions to authors

Complete this checklist by entering the page numbers from your manuscript where readers will find each of the items listed below.

Your article may not currently address all the items on the checklist. Please modify your text to include the missing information. If you are certain that an item does not apply, please write "n/a" and provide a short explanation.

Upload your completed checklist as an extra file when you submit to a journal.

In your methods section, say that you used the PRISMA-Preporting guidelines, and cite them as:

Moher D, Shamseer L, Clarke M, Ghersi D, Liberati A, Petticrew M, Shekelle P, Stewart LA. Preferred Reporting Items for Systematic Review and Meta-Analysis Protocols (PRISMA-P) 2015 statement. Syst Rev. 2015;4(1):1.

		Reporting Item	Page Number
**Title**			
Identification	#1a	Identify the report as a protocol of a systematic review	1
Update	#1b	If the protocol is for an update of a previous systematic review, identify as such	n/a

For peer review only

**Registration**			
	#2	If registered, provide the name of the registry (such as PROSPERO) and registration number	4
**Authors**			
Contact	#3a	Provide name, institutional affiliation, e-mail address of all protocol authors; provide physical mailing address of corresponding author	1-2
Contribution	#3b	Describe contributions of protocol authors and identify the guarantor of the review	2
**Amendments**			
	#4	If the protocol represents an amendment of a previously completed or published protocol, identify as such and list changes; otherwise, state plan for documenting important protocol amendments	n/a
**Support**			
Sources	#5a	Indicate sources of financial or other support for the review	2
Sponsor	#5b	Provide name for the review funder and / or sponsor	2
Role of sponsor or funder	#5c	Describe roles of funder(s), sponsor(s), and / or institution(s), if any, in developing the protocol	2
**Introduction**			

For peer review only

Rationale	#6	Describe the rationale for the review in the context of what is already known	5-7
Objectives	#7	Provide an explicit statement of the question(s) the review will address with reference to participants, interventions, comparators, and outcomes (PICO)	7
**Methods**			
Eligibility criteria	#8	Specify the study characteristics (such as PICO, study design, setting, time frame) and report characteristics (such as years considered, language, publication status) to be used as criteria for eligibility for the review	7-11
Information sources	#9	Describe all intended information sources (such as electronic databases, contact with study authors, trial registers or other grey literature sources) with planned dates of coverage	11-12
Search strategy	#10	Present draft of search strategy to be used for at least one electronic database, including planned limits, such that it could be repeated	12-13
Study records - data management	#11a	Describe the mechanism(s) that will be used to manage records and data throughout the review	13
Study records - selection process	#11b	State the process that will be used for selecting studies (such as two independent reviewers) through each phase of the review (that is, screening, eligibility and inclusion in meta-analysis)	13-14

For peer review only

Study records - data collection process	#11c	Describe planned method of extracting data from reports (such as piloting forms, done independently, in duplicate), any processes for obtaining and confirming data from investigators	14-15
Data items	#12	List and define all variables for which data will be sought (such as PICO items, funding sources), any pre-planned data assumptions and simplifications	15&30
Outcomes and prioritization	#13	List and define all outcomes for which data will be sought, including prioritization of main and additional outcomes, with rationale	15-16
Risk of bias in individual studies	#14	Describe anticipated methods for assessing risk of bias of individual studies, including whether this will be done at the outcome or study level, or both; state how this information will be used in data synthesis	16-17
Data synthesis	#15a	Describe criteria under which study data will be quantitatively synthesised	18-19
Data synthesis	#15b	If data are appropriate for quantitative synthesis, describe planned summary measures, methods of handling data and methods of combining data from studies, including any planned exploration of consistency (such as I2, Kendall's τ)	18-19
Data synthesis	#15c	Describe any proposed additional analyses (such as sensitivity or subgroup analyses, meta-regression)	19

For peer review only

Data synthesis	#15d	If quantitative synthesis is not appropriate, describe the type of summary planned	17
Meta-bias(es)	#16	Specify any planned assessment of meta-bias(es) (such as publication bias across studies, selective reporting within studies)	19-20
Confidence in cumulative evidence	#17	Describe how the strength of the body of evidence will be assessed (such as GRADE)	20

The PRISMA-P checklist is distributed under the terms of the Creative Commons Attribution License CC-BY 4.0. This checklist was completed on 02. December 2020 using https://www.goodreports.org/, a tool made by the EQUATOR Network in collaboration with Penelope.ai

For peer review only

BMJ Open

Effectiveness of interventions for people living with dementia and their carers in Chinese communities: protocol for a systematic review and meta-analysis of randomised controlled trials

Journal:	*BMJ Open*
Manuscript ID	bmjopen-2020-047560.R2
Article Type:	Protocol
Date Submitted by the Author:	25-Jun-2021
Complete List of Authors:	Shi, Cheng; The University of Hong Kong, Department of Social Work and Social Administration; Beijing Normal University, Center for Social Welfare Studies<br>Chen, Shuangzhou; The University of Hong Kong, Department of Social Work and Social Administration<br>Salcher-Konrad, Maximilian; London School of Economics and Political Science, Care Policy and Evaluation Centre (CPEC)<br>Choy, Jacky CP ; The University of Hong Kong, Department of Social Work and Social Administration<br>Luo, Hao; The University of Hong Kong, Department of Social Work and Social Administration; The University of Hong Kong, Department of Computer Science<br>Leung, Dara Kiu Yi; The University of Hong Kong, Social Work and Social Administration<br>Cai, Xinxin; The University of Hong Kong, Department of Social Work and Social Administration<br>Zeng, Yue; The University of Hong Kong, Department of Social Work and Social Administration<br>Dai, Ruizhi; The University of Hong Kong, Faculty of Social Sciences<br>Comas-Herrera, Adelina; London School of Economics and Political Science, Care Policy and Evaluation Centre (CPEC)<br>McDaid, David ; London School of Economics and Political Science, Care Policy and Evaluation Centre (CPEC)<br>Knapp, Martin; London School of Economics and Political Science, Care Policy and Evaluation Centre (CPEC)<br>Wong, Gloria; The University of Hong Kong, Department of Social Work and Social Administration
**Primary Subject Heading**:	Mental health
Secondary Subject Heading:	Health policy, Public health
Keywords:	Dementia < NEUROLOGY, Old age psychiatry < PSYCHIATRY, PUBLIC HEALTH, Health policy < HEALTH SERVICES ADMINISTRATION & MANAGEMENT

SCHOLARONE™
Manuscripts

I, the Submitting Author has the right to grant and does grant on behalf of all authors of the Work (as defined in the below author licence), an exclusive licence and/or a non-exclusive licence for contributions from authors who are: i) UK Crown employees; ii) where BMJ has agreed a CC-BY licence shall apply, and/or iii) in accordance with the terms applicable for US Federal Government officers or employees acting as part of their official duties; on a worldwide, perpetual, irrevocable, royalty-free basis to BMJ Publishing Group Ltd ("BMJ") its licensees and where the relevant Journal is co-owned by BMJ to the co-owners of the Journal, to publish the Work in this journal and any other BMJ products and to exploit all rights, as set out in our licence.

The Submitting Author accepts and understands that any supply made under these terms is made by BMJ to the Submitting Author unless you are acting as an employee on behalf of your employer or a postgraduate student of an affiliated institution which is paying any applicable article publishing charge ("APC") for Open Access articles. Where the Submitting Author wishes to make the Work available on an Open Access basis (and intends to pay the relevant APC), the terms of reuse of such Open Access shall be governed by a Creative Commons licence – details of these licences and which Creative Commons licence will apply to this Work are set out in our licence referred to above.

Other than as permitted in any relevant BMJ Author's Self Archiving Policies, I confirm this Work has not been accepted for publication elsewhere, is not being considered for publication elsewhere and does not duplicate material already published. I confirm all authors consent to publication of this Work and authorise the granting of this licence.

For peer review only

Title page

Effectiveness of interventions for people living with dementia and their carers in Chinese communities: protocol for a systematic review and meta-analysis of randomised controlled trials

Cheng Shi[1,2], Shuangzhou Chen[1], Maximilian Salcher-Konrad[3], Jacky CP Choy[1], Hao Luo,[1,4] Dara KY Leung[1], Xinxin Cai[1], Yue Zeng[1], Ruizhi Dai[5], Adelina Comas-Herrera[3], David McDaid[3], Martin Knapp[3], Gloria HY Wong[1]

Correspondence to Gloria Hoi Yan Wong, 524 The Jockey Club Tower, Centennial Campus, The University of Hong Kong, Pokfulam, Hong Kong. Email: ghywong@hku.hk

Author affiliations

[1]Department of Social Work and Social Administration, The University of Hong Kong, Hong Kong

[2]Center for Social Welfare Studies, Beijing Normal University, Beijing, China

[3]Care Policy and Evaluation Centre, London School of Economics and Political Science, London, UK

[4]Department of Computer Science, The University of Hong Kong, Hong Kong

[5]Faculty of Social Sciences, The University of Hong Kong, Hong Kong

Authors contact

Cheng Shi (cs2019@hku.hk), Shuangzhou Chen (chenshuangzhou@hku.hk), Maximilian Salcher-Konrad (m.salcher@lse.ac.uk), Jacky CP Choy (cpchoy@hku.hk), Hao Luo (haoluo@hku.hk), Dara KY Leung (daralky@hku.hk), Xinxin Cai (caixx@connect.hku.hk), Yue Zeng (yuezeng@connect.hku.hk), Ruizhi Dai (rdai@hku.hk), Adelina Comas-Herrera

For peer review only - http://bmjopen.bmj.com/site/about/guidelines.xhtml

For peer review only

(a.comas@lse.ac.uk), David McDaid (d.mcdaid@lse.ac.uk), Martin Knapp (m.knapp@lse.ac.uk), Gloria HY Wong (ghywong@hku.hk)

Author Contributions GHYW, CS, SC, JCPC, ACH, and MK defined the scope of the review and review question. GHYW, CS, SC, and MSK developed inclusion/exclusion criteria. HL designed the method for the Chinese bibliographical database search. GHYW, CS, SC, MSK, and DMD consulted on the bibliographical databases to be searched and search terms used. GHYW, CS, MSK, SC, JCPC, and DKYL provided information on methods of data synthesis. CS drafted the protocol. CS, SC, MSK, JCPC, HL, DKYL, XC, YZ, RD, ACH, DMD, MK, and GHYW contributed to the study design and reviewed the draft protocol. GHYW is the guarantor of the manuscript.

Funding This work is conducted as part of the 'Tools to Inform Policy: Chinese Communities Actions in Response to Dementia' (TIP-CARD) project, supported by the Hong Kong Research Impact Fund of the Research Grants Council (Project Reference No.: R7017-18). MSK, DMD, MK and ACH's contributions were supported by the UK Research and Innovation's Global Challenges Research Fund (ES/P010938/1) as part of the 'Strengthening Responses to Dementia in Developing Countries' (STRiDE) project. The funders were not involved in the development of this protocol.

Competing interests None declared

Patient consent for publication Not required.

Provenance and peer review Not commissioned; externally peer reviewed.

Word Count: 3965

For peer review only - http://bmjopen.bmj.com/site/about/guidelines.xhtml

Effectiveness of interventions for people living with dementia and their carers in Chinese communities: protocol for a systematic review and meta-analysis of randomised controlled trials

ABSTRACT

Introduction: As the largest and most rapidly ageing population, Chinese people are now the major driver of the continued growth in dementia prevalence globally. The need for evidence-based interventions in Chinese communities is urgent. Although a wide range of pharmacological and non-pharmacological interventions for dementia have been trialled in Chinese populations, the evidence has not been systematically synthesised. This systematic review and meta-analysis aim to map out the interventions for people living with dementia and their carers in Chinese communities worldwide and compare the effectiveness of these interventions.

Methods and analysis: This protocol followed the Preferred Reporting Items for Systematic Review and Meta-Analysis Protocols checklist. We will search Chinese (China National Knowledge Infrastructure, WanFang DATA) and English bibliographical databases (MEDLINE, EMBASE, PsycINFO, CINAHL Plus, Global Health, WHO Global Index Medicus, Virtual Health Library, Cochrane CENTRAL, Social Care Online, BASE, MODEM Toolkit, Cochrane Database of Systematic Reviews), complemented by hand searching of reference lists. We will include studies evaluating the effectiveness of interventions for dementia or mild cognitive impairment in Chinese populations, using a randomised control trial design, and published between January 2008 and June 2020. We will use a standardised form to extract data and version 2 of the Cochrane risk-of-bias tool for randomized trials to assess the risk of bias of the included studies. Collected data will be fully interpreted with narrative synthesis and analysed using pairwise and network meta-

For peer review only - http://bmjopen.bmj.com/site/about/guidelines.xhtml

analyses to pool intervention effects where sufficient information is available. We will perform subgroup analysis and meta-regression to explore potential reasons for heterogeneity.

Ethics and dissemination: No formal ethics approval is required for this protocol. The findings will facilitate the development of studies on interventions for dementia and timely inform dementia policymaking and practice. Planned dissemination channels include peer-reviewed publications, conference presentations, public events, and websites.

PROSPERO registration number: CRD42019134135

Strengths and limitations of this study

- This systematic review and meta-analysis will be the first review of randomised control trials (RCTs) on the effectiveness of both pharmacological and non-pharmacological interventions for people living with dementia and their carers in Chinese communities worldwide.
- We will use a comprehensive search strategy of publications in both Chinese bibliographical databases (China National Knowledge Infrastructure, WanFang DATA) and English bibliographical databases (MEDLINE, EMBASE, PsycINFO, CINAHL Plus, Global Health, WHO Global Index Medicus, Virtual Health Library, Cochrane CENTRAL, Social Care Online, BASE, MODEM Toolkit, Cochrane Database of Systematic Reviews).
- We will narratively synthesise the collected data to map out the dementia-related interventions studied in Chinese communities, and conduct pairwise and network meta-analyses to compare the effectiveness of interventions.
- This review will be limited by the number and quality of RCTs conducted in Chinese communities.

For peer review only - http://bmjopen.bmj.com/site/about/guidelines.xhtml

INTRODUCTION

Around 50 million people currently live with dementia worldwide, of whom 20 per cent are Chinese populations.[1] Chinese populations refer to people of Chinese ethnicity or national heritage, regardless of their nationality or region of residence. As the largest and most rapidly ageing population, the Chinese are now the major driver in the continued growth of global dementia prevalence.[2] Due to the physical and emotional challenges involved in caring, dementia affects not only people living with the condition, but also their families, formal carers, and other supporters.[3] With a culture emphasising filial piety, coupled with insufficient care services, family care is often the main supporting resource for people living with dementia (PLwD) in Chinese communities worldwide. Dementia has been recognised as one of the most burdensome diseases among Chinese populations.[4]

There is currently no cure for dementia, although symptoms can be managed with effective intervention and good care.[3] China recently launched its national dementia strategy, one of whose main tasks is to improve the wellbeing of PLwD by increasing service provision.[5] Taiwan updated its dementia policy in 2017, promoting dementia research, innovation, and development as one of its seven strategies.[6] In Macau's 10-year Plan of Action on Dementia Services published in 2016, strengthening community services and caregiver support comprises one of its five strategies.[7] In Hong Kong, a government services review and programme plan published in 2017[8,9] highlighted the need to strengthen services for PLwD and recommended a seven-stage model for dementia service following the World Health Organization and Alzheimer's Disease International's framework.[10] The need for evidence-based interventions and care services in Chinese populations is urgent.

For peer review only - http://bmjopen.bmj.com/site/about/guidelines.xhtml

Studies on dementia interventions appear to be scarce in Asian populations.[2] Most evidence on drug treatment and non-pharmacological interventions has been generated in Western countries, with questionable relevance for Chinese populations. For example, Cognitive Stimulation Therapy (CST) used alone or in combination with medication was shown to be effective and even cost-effective in improving cognition and quality of life, [11-14] leading to a recommendation for routine use by England's National Institute for Health and Care Excellence (NICE),[15] and by Alzheimer's Disease International.[16] In contrast, preliminary findings from a study applying CST with Hong Kong Chinese suggest that a larger number of participants needed to be treated to achieve clinically significant improvement in cognition.[17] Such discrepancies in an intervention's effect, possibly due to cultural differences, highlight the importance of generating evidence on the effectiveness of dementia-related interventions relevant to local populations.

There is now increasing evidence on a wide range of interventions for dementia undertaken in Chinese populations. A few reviews have been published, focusing on specific interventions and subtypes of dementia, such as the efficacy of donepezil in Chinese with Alzheimer's disease,[18] Chinese herbal medicine as adjunctive therapy for vascular dementia,[19] and traditional Chinese mind-body exercise (baduanjin) in older adults with mild cognitive impairment (MCI).[20] Growing evidence also suggests that the therapeutic response to dementia intervention (e.g., donepezil) might differ between Chinese and Western populations due to pharmacogenetic factors,[18,21] thus emphasising the need for more accurate evaluations of interventions tailored to Chinese populations.[22]

Some existing and ongoing studies aim to synthesise evidence for dementia intervention and care, including the Modelling Outcome and Cost Impacts of Interventions for Dementia (MODEM) project[23] with a dementia evidence toolkit (https://www.modem-dementia.org.uk/)

For peer review only - http://bmjopen.bmj.com/site/about/guidelines.xhtml

covering dementia interventions in English literature, and the Strengthening Responses to Dementia in Developing Countries (STRiDE) project (https://stride-dementia.org/) with an ongoing systematic review and meta-analysis on the evidence in seven low- and middle-income countries.[24] There is no comprehensive evidence synthesis on the effectiveness of dementia or dementia-related interventions that cover different types of dementia (e.g., Alzheimer's disease, vascular dementia, frontotemporal dementia, Lewy body disease and mixed dementia) and interventions (e.g., pharmacological treatment, psychosocial intervention, and traditional Chinese medicine) conducted in Chinese populations. Existing systematic reviews have focused mainly on the English language literature, where evidence from high-income areas such as Hong Kong and Taiwan can be found. Although Chinese academic databases have been recognised as a valuable resource for dementia-related studies, they have not been fully explored.[25-27]

To our knowledge, this will be the first systematic review and meta-analysis to comprehensively synthesise and assess the evidence on the effectiveness of interventions for PLwD and their carers among Chinese populations in Chinese and English bibliographical databases. We aim to 1) map out interventions for dementia studied in Chinese communities, and 2) compare the effectiveness of those interventions for achieving desired outcomes. This study will contribute to shape understanding of existing evidence on effectiveness of dementia-related interventions, improve quality of life of PLwD and their carers, and provide valuable information for practice, policymaking and further research. As part of a research project, Tools to Inform Policy: Chinese Communities Actions in Response to Dementia (TIP-CARD; www.tip-card.hku.hk/), this study also aligns with the above-mentioned dementia evidence synthesis effort by the STRiDE project.[24]

For peer review only - http://bmjopen.bmj.com/site/about/guidelines.xhtml

METHODS AND ANALYSIS

Protocol and registration

This protocol for this systematic review and meta-analysis followed the Preferred Reporting Items for Systematic Review and Meta-Analysis Protocols checklist.[28] This study has been registered on the PROSPERO platform (www.crd.york.ac.uk/prospero; registration number: CRD42019134135).

For peer review only

Eligibility criteria

Population

We will include studies conducted among adults (aged 18 years and over) living with dementia or MCI and their carers in Chinese populations. We will include relevant studies conducted in any type of care settings, such as home, community, residential homes, clinics, hospitals, and other care settings. Participant characteristics such as gender, education, and age at diagnosis will not be used for excluding studies.

We will include studies covering people living with any type and stage of dementia. Dementia, as a major neurocognitive disorder, describes a group of symptoms of cognitive decline, including, but not limited to, Alzheimer's disease, vascular dementia, frontotemporal dementia, Lewy body disease, and mixed dementia. Studies conducted among people living with MCI, mild neurocognitive disorder, vascular cognitive impairment, and no dementia (VCIND) will be eligible for inclusion due to the higher risk of developing dementia in later years.[29] We will also include studies conducted among people with diseases co-occurring with dementia or MCI, and people with dementia or MCI with unknown subtype, as long as the diagnostic criteria for dementia or MCI were explicated.

For peer review only - http://bmjopen.bmj.com/site/about/guidelines.xhtml

Our definition of dementia carer refers to persons involved in care provision and management and will not depend on whether or not the carer is paid, lives with the person they care for, or provide direct or indirect care. Therefore, dementia carers include health and social care professionals, care managers, care workers, administrative staff of care facilitators, family carers, other unpaid carers, and family members assisting with care decisions. We will focus on studies conducted among people of Chinese ethnicity or national heritage regardless of their nationality or location of residence. Studies without explicating the proportion of Chinese participants over 50% or studies without a specific subgroup analysis for Chinese participants will be excluded.

Intervention

Based on the effectiveness perspective,[24,30] any type of interventions for improving desired outcomes will be eligible. We will include studies on pharmacological treatment, non-pharmacological intervention (e.g., cognitive intervention, technological intervention, training, and exercise), or multicomponent interventions. We will exclude studies: 1) where no clear intervention was described, 2) on primary prevention of dementia, and 3) on non-interventional studies.

Comparison

Given the broad range for interventions of interest, any comparisons within the context of eligible study design will be acceptable for inclusion, such as active comparators, treatment as usual, placebo, and no treatment.

For peer review only - http://bmjopen.bmj.com/site/about/guidelines.xhtml

Outcomes

Any type of outcomes of dementia-related intervention will be eligible for inclusion from the perspective of effectiveness, which may affect individuals, families, the dementia care workforce, wider society, and social or health care systems. Dementia often triggers complex problems in many domains.[22] According to the MODEM dementia evidence toolkit (https://www.modem-dementia.org.uk/), outcomes measured in existing studies may include 1) cognition, behavioural and psychological symptoms, functional status, physical health and quality of life of PLwD; 2) carer burden, carer's mental health, quality of life and other carer outcomes (e.g., financial burdens); 3) service use, cost reduction (including hospital use reduction and care home admission delay) and service satisfaction; 4) risk reduction (of dementia and comorbidities) and prevention or management of comorbidities. To capture the diversity of interventions trialled in Chinese communities, we will accept all outcome measures that reflect intervention effectiveness.

Study design

To identify potential causal relationships, we will only include studies using randomised controlled trial (RCT) or cluster RCT designs. To control study quality, we will only include RCTs with a low risk of bias in the process of evidence generation. According to version 2 of the Cochrane risk-of-bias tool for randomized trials (RoB 2), [31] methods used for generating random allocation sequence indicating low risk of bias include computer-generated random numbers, a random number table, coin tossing, shuffling cards or envelopes, throwing dice, or drawing lots. Studies

For peer review only - http://bmjopen.bmj.com/site/about/guidelines.xhtml

that use no random element or provide no information on the generation process of the random allocation sequence will be excluded.

To minimise small-study effects,[32,33] we will exclude studies with a sample size of less than 50 in either the intervention group or comparison group(s) for the eligible population. For studies conducted with a population of mixed ethnicity, the sample size of each study arm for Chinese sub-group analysis should be greater than 50 participants. For studies in which more than 50% of participants are Chinese and all participants are randomly grouped, the sample size of each study arm is expected to be greater than 50 participants regardless of ethnicity.

Publication type

We will include the primary publications of intervention studies and grey literature evaluating the effectiveness of dementia-related interventions in Chinese populations. Relevant systematic reviews or scoping reviews will be included in the first step screening, and then will be used to complement the primary publications by hand searching of reference lists. Conference abstracts will be included if they contain sufficient information to assess eligibility for inclusion.

Publication period

Studies published between January 2008 and June 2020.

Language

Studies will be limited to English and Chinese language publications.

For peer review only - http://bmjopen.bmj.com/site/about/guidelines.xhtml

Information sources

We will search two major Chinese bibliographical databases (China National Knowledge Infrastructure and WanFang DATA) and English bibliographical databases (MEDLINE, EMBASE, PsycINFO, CINAHL Plus, Global Health, WHO Global Index Medicus, Virtual Health Library, Cochrane CENTRAL, Social Care Online, BASE, MODEM Toolkit, Cochrane Database of Systematic Reviews). Hand searching of reference lists among review studies will complement the database searches.

Search strategy

We will adapt an established search strategy protocol[24] used to search for English language literature. Corresponding Chinese search terms will be translated and adapted by three bilingual researchers (GHYW, SC, and CS) experienced in dementia/ageing research with a training background in psychology, psychiatry, translation, social work, and social policy from Hong Kong and mainland China. Search terms in English and Chinese are listed in Table 1. In studies published in English, the search terms related to Chinese populations include 'China', 'Chinese', 'Sino', 'Hong Kong', 'Taiwan', 'Taiwanese', 'Macau', and 'Asian'.

For studies published in English, we will first extract eligible study records identified from an ongoing systematic review,[24] which used the same search strategy and search terms for dementia intervention and covered studies published between 2008 and 2018. Then we will search these terms for Chinese populations in the title, abstract, and keywords. Second, we will repeat the English bibliographical database search mentioned above to identify studies published between January 2019 and June 2020.

For peer review only - http://bmjopen.bmj.com/site/about/guidelines.xhtml

For studies published in Chinese, we will use Python,[34] a programming language, to facilitate Chinese bibliographical database searching by using dementia-related search terms (search items #1-4 in Table 1). This is because of the technical challenge posed by limitations on the number of search terms and exported records per time in the two Chinese bibliographical databases. The search results for dementia-related study records will be exported in a Microsoft Excel spreadsheet. Then we will search the intervention-related terms (search items #5-53) in the title and abstract.

Study records

Data management

To deal with a potentially large number of search results and various data sources in two languages, we will manage references using two web-based software packages during the review and extraction process: 1) Rayyan (https://rayyan.qcri.org/), a web and mobile app that can facilitate the initial screening of abstracts and titles using a semi-automated process,[35] and 2) Covidence (https://www.covidence.org/), an internet-based software platform for managing systematic reviews, including study selection, risk of bias assessment, and data extraction.

Duplicate publications will be checked based on title, author, journal, and year using Microsoft Excel, and the 'Find duplicates' function in Rayyan and Covidence. Multiple publications from the same study will be identified based on the key information (e.g., authors' names, study design, intervention, and outcomes) from the full-texts, or by contacting authors for clarification if needed. Once confirmed, included multiple publications will be linked on Covidence.

For peer review only - http://bmjopen.bmj.com/site/about/guidelines.xhtml

Study selection

Study selection will be a two-step process, with detailed explanations for inclusion and exclusion criteria in each step. First, two researchers will independently screen the title and abstract and determine the study's inclusion or exclusion on Rayyan. A justification (criterion) will be required for any exclusion decision. Studies with insufficient information in the title and abstract to enable a decision to be made will be included at this stage. The Rayyan machine learning-based classifier[35] will be considered to facilitate the title and abstract screening, given the potentially work overload. Using a certain number of manually screened studies as a training dataset, Rayyan will generate a relevance rating for each study, ranging from 0.5 (lowest) to 5 (highest).[35] We may use a low relevance score (e.g., below 1.5) as a threshold to guide study exclusion.

Second, studies included after title and abstract screening will be uploaded to Covidence for full-text review by two independent reviewers, who will provide a justification for each excluded study. Review studies will be excluded at this stage, although their reference lists will be used to complement the database search results.

All disagreements in each step will be resolved through discussion between the two reviewers. If consensus is unreachable, a third reviewer will be consulted for a final decision.

Reviewers for title and abstract screening and full-text review will be able to read and understand inclusion/exclusion criteria for publications in both English and simplified Chinese.

Upon completion of the selection process, we will generate a Preferred Reporting Items for Systematic Reviews and Meta-Analyses flowchart[36] to illustrate the inclusion and exclusion of studies at each stage in study selection.

Data collection process

For peer review only - http://bmjopen.bmj.com/site/about/guidelines.xhtml

We will use a standardised form based on the template available from Covidence for data extraction that will be pilot-tested using included studies. To ensure data consistency across reviewers, we will organise exercises and group discussions for reviewer training. Due to the anticipated large number of potentially eligible studies, the data extraction form will be completed by one reviewer and verified by the second reviewer. We will keep all records of corrections or amendments to the data extraction. For studies that do not report the required information, we will contact the authors to request information.

We will prepare the data extraction form in English. For studies in Chinese, our bilingual reviewers will complete data extraction using the original expression in Chinese full-texts except for the outcome name and brief introduction of the intervention, which will be recorded in English based on the English abstract if available or manual translation. The final extracted evidence from both the English and Chinese studies will be verified by one bilingual researcher (CS) to ensure consistent translation.

Data items

We will extract information on items listed in Box 1 from the included studies.

Outcomes and prioritisation

In line with our research aims, we will first record all types of outcome and outcome measures stated in the included studies to map out the dementia-related interventions conducted in Chinese communities. Due to the anticipated number of Chinese studies from an ongoing review,[24] we will prioritise the following outcomes of interest when extracting outcome results from included studies.

For peer review only - http://bmjopen.bmj.com/site/about/guidelines.xhtml

For peer review only

As dementia is a condition affecting cognition by definition, we will prioritise outcome on changes in cognition. Common assessments for measuring cognitive impairment level or performance include the Mini–Mental State Examination (MMSE), the Montreal Cognitive Assessment (MoCA), and the Alzheimer's Disease Assessment Scale-Cognitive Subscale (ADAS-Cog). Given the effects of dementia on the ability to organise activities,[22] we will also focus on changes in functional ability following treatment. For example, the Disability Assessment for Dementia (DAD) is designed for evaluating functional ability to complete activities of daily living (ADL) and instrumental activities of daily living (IADLs) among PLwD.

Caring for a person living with dementia can be very stressful, which may lead to a higher level of depression or health issues.[37] For studies conducted among carers of PLwD, we will focus on changes in quality of life and carer burden,[38,39] as measured by tools such as the EUROHIS-QOL 8-item index and the Zarit Burden Interview (ZBI), respectively.

If the outcome of interest or its measure is not reported in included studies, we will extract the outcome results that are reported as the primary outcome in the original included study. Where feasible, we will also be open to examining other outcomes evaluated in the included studies.

We will extract results of outcomes of interest measured at each time point reported in included studies. Nevertheless, we will afford preference to the endpoint of the study in the main data synthesis. Results at multiple time points will be used for subgroup analysis and meta-regression to explore the short-term and long-term effects of outcomes.

Risk of bias in individual studies

We will use the Cochrane Collaboration's recently updated risk-of-bias tool (RoB 2)[31] to assess the quality of included studies in Covidence. Two reviewers will make independent judgements

For peer review only - http://bmjopen.bmj.com/site/about/guidelines.xhtml

based on the criteria for judging the risk of bias (RoB). Disagreement will be resolved by discussion and arbitrated by a third reviewer if consensus is unreachable. For studies that do not provide sufficient information in full-texts for RoB assessment, we will search for the study's protocol, trial registry information, or other relevant materials to facilitate the judgement. The absence of a pre-specified analysis plan may raise some concerns in the domain for bias in selection of the reported result.

Data synthesis

Evidence on dementia-related interventions in PLwDs and carers will be analysed separately. Studies of family-based or dyadic interventions involving both PLwDs and carers will be categorised according to the subject of each outcome.

Narrative synthesis

To map dementia-related interventions conducted in Chinese communities, we will undertake a narrative synthesis to fully interpret the extracted evidence from all included studies. We will first describe and summarise disease characteristics, features of the intervention, number of participants, participant characteristics, outcomes, outcome measures, and indication of risk of bias assessment in a tabular form. In line with the Guidance on the Conduct of Narrative Synthesis in Systematic Reviews,[40] we will then explore the relationship among types of interventions (or details of pharmacological, non-pharmacological and multicomponent interventions), outcomes, and outcome measures conducted in Chinese populations. Idea webbing will be used to visually describe conceptual linkages through examination of extracted data if feasible. The key questions here are what (types of) interventions have been conducted in Chinese communities, what specific

For peer review only - http://bmjopen.bmj.com/site/about/guidelines.xhtml

outcomes those interventions target and what measures are used for those outcomes. We expect to identify research gaps in this field for future studies and practices.

Meta-analyses

To compare the effectiveness of interventions for outcomes of interest (described in the 'Outcome and prioritisation' section), we will conduct quantitative synthesis of treatment effects through meta-analyses where sufficient information is available. For a specific outcome, we will perform a series of pairwise meta-analyses for all direct comparisons (e.g., one comparison between an intervention group and a control or another intervention group).[41] Due to the underlying difference between studies in terms of participants, intervention details and care settings, a random-effects pooling model will be conducted by default for an overall summary estimate by weighting studies using a combination of within- and between-study variance. When the included studies use different instruments to evaluate the same outcome (e.g., MMSE, MoCA, and ADAS-Cog for measuring cognition), we will use standardised mean difference (the absolute mean difference between the intervention group and control group divided by the standard deviation in the control group) for continuous outcomes and relative risks for dichotomous outcomes to compute the effect size for each study.

To compare the effectiveness for multiple interventions, we will use network meta-analysis to combine direct and indirect evidence for relevant treatment effects.[42] In network meta-analyses, different comparisons among two or more of the treatments can be included in one analysis. We will generate network geometry to visualise and assess the treatment networks, and estimate and combine comparative effects from direct and indirect evidence. In examining the transitivity hypothesis of network meta-analysis, we will use 'loop-specific approaches' to detect the inconsistency of a network of interventions, including local inconsistency test to evaluate the

For peer review only - http://bmjopen.bmj.com/site/about/guidelines.xhtml

loop inconsistency in regions of network separately[43] and global inconsistency test to evaluate the incoherence in the overall network.[44]

Sensitivity analyses will be conducted to explore the robustness of the meta-analysis results by varying the analytic data or methods, including analysing studies only with a low risk of bias and trials using a placebo as a comparator.[45]

Dealing with missing data

When there are missing data, we will attempt to obtain these by contacting the study author(s). If unsuccessful, we will consider using imputation methods to impute the missing value[46] or exclude studies with missing data from the quantitative analysis. We will use sensitivity analysis to evaluate the potential influence on the overall treatment effects of included studies that use per-protocol analysis or suggest the result was biased by missing outcome data (i.e., high risk of bias) based on the RoB 2 assessment tool.[31]

Subgroup analysis and meta-regression

We will calculate Cochrane's Q statistic and the I^2 statistic to estimate the heterogeneity of the included studies.[47] If statistical heterogeneity is observed, we will conduct subgroup analysis and meta-regression to explore the potential reasons for the differences. Potential candidate covariates for subgroup analysis and meta-regression include intervention characteristics (e.g., types of intervention, intervention dosage, and duration), participant characteristics (e.g., age, gender, education, severity of dementia, and type of dementia), care settings, follow-up period

For peer review only - http://bmjopen.bmj.com/site/about/guidelines.xhtml

(e.g., at three, six, and 12 months) and locations (e.g., mainland China, Hong Kong, Taiwan, Macau, and other Chinese communities worldwide).

Meta-bias(es)

For each meta-analysis, we will use a funnel plot asymmetry assessment to detect meta-biases. Statistical tests for funnel plot asymmetry will be performed when at least ten studies included in the meta-analysis.[48] Contour lines indicating various statistical significance will be used to aid visual interpretation of funnel plots. If funnel plot asymmetry is observed, we will also consider other possible reasons apart from non-reporting bias such as poor methodological quality and true heterogeneity of the included studies.[49]

Confidence in cumulative estimate

We will use the Grading of Recommendations Assessment, Development and Evaluation (GRADE) approach[50] to assess the quality of evidence. The domains of the assessment include risk of bias, inconsistency, indirectness of evidence, imprecision, and publication bias.

Patient and public involvement

Neither patients nor the public will be involved in the design or development of this review protocol. However, stakeholders, including PLwD, family members, care staff, healthcare professionals, and policymakers, will be engaged in the dissemination plan as described below.

ETHICS AND DISSEMINATION

For peer review only - http://bmjopen.bmj.com/site/about/guidelines.xhtml

This protocol for a systematic review and meta-analysis describes the methods to identify and synthesise published evidence on the effectiveness of interventions for PLwD and their carers in Chinese communities. No formal ethics approval is required for this protocol. The findings from this study will facilitate the development of studies on interventions for dementia and provide timely information for dementia policymaking and practice. We will target both professionals and non-specialist audiences in disseminating the outcomes of the review through print and events, including peer-reviewed publications, conference presentations, public events, and publicly accessible websites.

REFERENCES

1. Patterson C. World Alzheimer Report 2018: The State of the Art of Dementia Research: New Frontiers. London: Alzheimer's Disease International, 2018.

2. Prince M, Wimo A, Guerchet M, et al. World Alzheimer Report 2015: The Global Impact of Dementia. London: Alzheimer's Disease International, 2015.

3. Livingston G, Sommerlad A, Orgeta V, et al. Dementia prevention, intervention, and care. *Lancet* 2017;390(10113):2673-734. doi: 10.1016/S0140-6736(17)31363-6

4. Lou VWQ, Chi I. Is China Ready For Dementia. *Alzheimers and Neurodegenerative Diseases* 2020:6(1/2): 041. doi: 10.24966/AND-9608/100041

5. National Health Commission. Work Plan on Exploring Special Services for Senile Dementia Prevention and Treatment (Tansuo laonian chidai fangzhi tese fuwu gongzuo fang'an), 2020.

6. Ministry of Health and Welfare, Republic of China (Taiwan),. Taiwan Dementia Policy: A Framework for Prevention and Care 2.0, 2017.

For peer review only - http://bmjopen.bmj.com/site/about/guidelines.xhtml

7. Government of Macao SAR. 10-Year Plan of Action on Services for Older Persons 2016-2025. Macao Special Administrative Region Government, 2016.

8. Food and Health Bureau. Mental Health Review Report. Hong Kong: Food and Health Bureau, Hong Kong Special Administrative Region Government, 2017.

9. Working Group on Elderly Services Programme Plan, Elderly Commission of Government of Hong Kong SAR,. Elderly Services Programme Plan, 2017.

10. World Health Organization. Dementia: A Public Health Priority. Geneva: World Health Organization, 2012.

11. Knapp M, Thorgrimsen L, Patel A, et al. Cognitive stimulation therapy for people with dementia: cost-effectiveness analysis. *Brit J Psychiat* 2006;188:574-80. doi: 10.1192/bjp.bp.105.010561

12. Woods B, Aguirre E, Spector AE, et al. Cognitive stimulation to improve cognitive functioning in people with dementia. *Cochrane Db Syst Rev* 2012(2) doi: 10.1002/14651858.CD005562.pub2

13. Huntley JD, Gould RL, Liu K, et al. Do cognitive interventions improve general cognition in dementia? A meta-analysis and meta-regression. *Bmj Open* 2015;5(4) doi: 10.1136/bmjopen-2014-005247

14. D'Amico F, Rehill A, Knapp M, et al. Maintenance Cognitive Stimulation Therapy: An Economic Evaluation Within a Randomized Controlled Trial. *J Am Med Dir Assoc* 2015;16(1):63-70. doi: 10.1016/j.jamda.2014.10.020

15. National Institute for Health and Care Excellence. Dementia: Assessment, Management and Support for People Living with Dementia and their Carers. London: NICE, 2018.

For peer review only - http://bmjopen.bmj.com/site/about/guidelines.xhtml

16. Alzheimer's Disease International. World Alzheimer Report 2011: The Benefits of Early Diagnosis and Intervention: London: Alzheimer's Disease International, 2011.

17. Wong GHY, Yek OPL, Zhang AY, et al. Cultural adaptation of cognitive stimulation therapy (CST) for Chinese people with dementia: multicentre pilot study. *Int J Geriatr Psych* 2018;33(6):841-48. doi: 10.1002/gps.4663

18. Zhang N, Gordon ML. Clinical efficacy and safety of donepezil in the treatment of Alzheimer's disease in Chinese patients. *Clin Interv Aging* 2018;13:1963-70. doi: 10.2147/CIA.S159920

19. Zeng LF, Zou YP, Kong LS, et al. Can Chinese herbal medicine adjunctive therapy improve outcomes of senile vascular dementia? Systematic review with meta-analysis of clinical trials. *Phytother Res* 2015;29(12):1843-57. doi: 10.1002/ptr.5481

20. Xia R, Qiu PT, Lin HY, et al. The effect of traditional Chinese mind-body exercise (Baduanjin) and brisk walking on the dorsal attention network in older adults with mild cognitive impairment. *Front Psychol* 2019;10:2075 doi: ARTN 207510.3389/fpsyg.2019.02075

21. Liu HC, Hong CJ, Wang SJ, et al. ApoE genotype in relation to AD and cholesterol - A study of 2,326 Chinese adults. *Neurology* 1999;53(5):962-66. doi: Doi 10.1212/Wnl.53.5.962

22. Livingston G, Huntley J, Sommerlad A, et al. Dementia prevention, intervention, and care: 2020 report of the Lancet Commission. *Lancet* 2020;396(10248):413-46. doi: 10.1016/S0140-6736(20)30367-6

23. Comas-Herrera A, Knapp M, Wittenberg R, et al. MODEM: A comprehensive approach to modelling outcome and costs impacts of interventions for dementia. Protocol paper. *BMC Health Serv Res* 2017;17(1):25. doi: 10.1186/s12913-016-1945-x

For peer review only - http://bmjopen.bmj.com/site/about/guidelines.xhtml

24. Salcher-Konrad M, Naci H, McDaid D, et al. Effectiveness of interventions for dementia in low- and middle-income countries: protocol for a systematic review, pairwise and network meta-analysis. *BMJ Open* 2019;9(6):e027851. doi: 10.1136/bmjopen-2018-027851

25. Fung ICH. Chinese journals: a guide for epidemiologists. *Emerg Themes Epidemi* 2008;5:20 doi: 10.1186/1742-7622-5-20

26. Xia J, Wright J, Adams CE. Five large Chinese biomedical bibliographic databases: accessibility and coverage. *Health Info Libr J* 2008;25(1):55-61. doi: 10.1111/j.1471-1842.2007.00734.x

27. Chan KY, Wang W, Wu JJ, et al. Epidemiology of Alzheimer's disease and other forms of dementia in China, 1990-2010: a systematic review and analysis. *Lancet* 2013;381(9882):2016-23. doi: 10.1016/S0140-6736(13)60221-4

28. Shamseer L, Moher D, Clarke M, et al. Preferred reporting items for systematic review and meta-analysis protocols (PRISMA-P) 2015: elaboration and explanation. *BMJ* 2015;350:g7647. doi: 10.1136/bmj.g7647 [published Online First: 2015/01/04]

29. Mitchell AJ, Shiri-Feshki M. Rate of progression of mild cognitive impairment to dementia–meta-analysis of 41 robust inception cohort studies. *Acta Psychiatrica Scandinavica* 2009;119(4):252-65.

30. Reeves BC, Deeks JJ, Higgins J. 13 Including non-randomized studies. *Cochrane handbook for systematic reviews of interventions* 2008;1:391.

31. Sterne JAC, Savovic J, Page MJ, et al. RoB 2: a revised tool for assessing risk of bias in randomised trials. *BMJ* 2019;366:l4898. doi: 10.1136/bmj.l4898 [published Online First: 2019/08/30]

For peer review only - http://bmjopen.bmj.com/site/about/guidelines.xhtml

32. Chen HY, Shen DT, Ji DZ, et al. Prevalence and burden of hepatitis D virus infection in the global population: a systematic review and meta-analysis. *Gut* 2019;68(3):512-21. doi: 10.1136/gutjnl-2018-316601 [published Online First: 2018/09/20]

33. Turner RM, Bird SM, Higgins JP. The impact of study size on meta-analyses: examination of underpowered studies in Cochrane reviews. *PLoS One* 2013;8(3):e59202. doi: 10.1371/journal.pone.0059202 [published Online First: 2013/04/02]

34. Python Software Foundation. Python language reference, version 3.6. 8. 2020

35. Ouzzani M, Hammady H, Fedorowicz Z, et al. Rayyan—a web and mobile app for systematic reviews. *Syst Rev* 2016;5(1):210. doi: 10.1186/s13643-016-0384-4

36. Moher D, Liberati A, Tetzlaff J, et al. Preferred Reporting Items for Systematic Reviews and Meta-Analyses: The PRISMA Statement. *PLOS Medicine* 2009;6(7):e1000097. doi: 10.1371/journal.pmed.1000097

37. Baumgarten M, Battista RN, Infanterivard C, et al. The psychological and physical health of family members caring for an elderly person with dementia. *J Clin Epidemiol* 1992;45(1):61-70. doi: Doi 10.1016/0895-4356(92)90189-T

38. Chien WT, Lee IYM. Randomized controlled trial of a dementia care programme for families of home-resided older people with dementia. *J Adv Nurs* 2011;67(4):774-87. doi: 10.1111/j.1365-2648.2010.05537.x

39. Lam LCW, Lee JSW, Chung JCC, et al. A randomized controlled trial to examine the effectiveness of case management model for community dwelling older persons with mild dementia in Hong Kong. *Int J Geriatr Psych* 2010;25(4):395-402. doi: 10.1002/gps.2352

40. Popay J, Roberts H, Sowden A, et al. Guidance on the conduct of narrative synthesis in systematic reviews. *A product from the ESRC Methods Programme* 2006;1:b92.

For peer review only - http://bmjopen.bmj.com/site/about/guidelines.xhtml

For peer review only

41. Lau J, Ioannidis JP, Schmid CH. Quantitative synthesis in systematic reviews. *Ann Intern Med* 1997;127(9):820-6. doi: 10.7326/0003-4819-127-9-199711010-00008 [published Online First: 1998/02/12]

42. Cuijpers P, Noma H, Karyotaki E, et al. Effectiveness and acceptability of cognitive behavior therapy delivery formats in adults with depression: a network meta-analysis. *JAMA Psychiatry* 2019;76(7):700-07. doi: 10.1001/jamapsychiatry.2019.0268

43. Chaimani A, Higgins JPT, Mavridis D, et al. Graphical Tools for Network Meta-Analysis in STATA. *Plos One* 2013;8(10) doi: 10.1371/journal.pone.0076654

44. Higgins JPT, Jackson D, Barrett JK, et al. Consistency and inconsistency in network meta-analysis: concepts and models for multi-arm studies. *Res Synth Methods* 2012;3(2):98-110. doi: 10.1002/jrsm.1044

45. Centre for Reviews and Dissemination. CRD's guidance for undertaking reviews in healthcare: CRD, University of York 2009.

46. Crameri A, von Wyl A, Koemeda M, et al. Sensitivity analysis in multiple imputation in effectiveness studies of psychotherapy. *Front Psychol* 2015;6 doi: 10.3389/fpsyg.2015.01042

47. Higgins JP, Thompson SG. Quantifying heterogeneity in a meta-analysis. *Stat Med* 2002;21(11):1539-58. doi: 10.1002/sim.1186 [published Online First: 2002/07/12]

48. Sterne JA, Sutton AJ, Ioannidis JP, et al. Recommendations for examining and interpreting funnel plot asymmetry in meta-analyses of randomised controlled trials. *Bmj* 2011;343

49. Egger M, Smith GD, Schneider M, et al. Bias in meta-analysis detected by a simple, graphical test. *Bmj* 1997;315(7109):629-34.

50. Schünemann H, Brożek J, Guyatt G, et al. Handbook for Grading the Quality of Evidence and the Strength of Recommendations using the GRADE Approach: Cochrane Collaboration;

For peer review only - http://bmjopen.bmj.com/site/about/guidelines.xhtml

2013 [Available from: https://gdt.gradepro.org/app/handbook/handbook.html accessed on 19 Oct 2020.

For peer review only

For peer review only - http://bmjopen.bmj.com/site/about/guidelines.xhtml

TABLES

Table 1 Search terms related to dementia and intervention in English and Simplified Chinese

Search #	Search terms in English	Search terms in Simplified Chinese
1	Dementia	痴呆 or 失智 or 认知症
2	Cognitive disorder	认知障碍 or 认知功能障碍 or 认知紊乱 or 认知功能紊乱
3	Alzheimer	茨海默 or 兹海默
4	((cognit* or memory or cerebr*) adj3 (impair* or los* or declin* or deteriorat* or degenerat*)).mp.	(认知 or 记忆 or 脑) (缺损 or 缺失 or 退*化 or 衰退 or 下降 or 损伤 or 恶化 or 损害 or 退行)
5	(Intervention* or therap* or treatment* or program* or manage* or prevent* or diagnos* or polic*).mp.	干预 or 介入 or 治疗 or 疗法 or 方案 or 处理 or 预防 or 诊断 or 措施 or 手段 or 政策 or 应用 or 支持 or 效果 or 疗效 or 观察 or 价值 or 临床 or 分析
6	Cognitive therapy	认知 (治疗 or 疗法)
7	Cognitive stimulation	认知 (刺激 or 促进)
8	Cognitive training	认知训练
9	Cognitive rehabilitation	认知 (复康 or 复健 or 康复)
10	Drug therapy or pharmacotherapy	*药*
11	Cholinesterase inhibitors	胆碱分解抑制剂 or 胆碱酵素抑制剂 or 胆碱酶抑制剂
12	Cholinesterase agent	胆碱分解剂 or 胆碱酵素剂 or 胆碱酶剂
13	(Sedative or tranquili* adj3 (agent* or drug*)).mp.	(镇静 or 镇定 or 安神 or 安定) (药 or 剂)
14	Antipsychotic or neuroleptic (agent* or drug*)	抗精神病 (药 or 剂)
15	exp Serotonin Reuptake Inhibitors or ssri	(血清素 or 5-羟色胺) (再摄取 or 再吸收 or 回收) 抑制剂
16	Benzodiazepines	苯二氮平 or 苯二氮卓
17	(memantine or donepezil or rivastigmine or galantamine or souvenaid or risperidone or haloperidol or olanzapine	(美金刚 or 美金胺) (多奈呱齐 or 多奈呱其) (卡巴拉汀 or 利斯的明) (加兰他敏 or 加兰他明 or 格兰他明) (智敏捷) (利培酮 or 利螺环酮) (氟哌啶醇 or 氟哌丁苯 or 氟哌醇 or 卤吡醇) (奥氮平) (喹硫平) (西酞普兰) (右美沙芬 or 右旋美沙酚 or 右旋美索芬 or 右甲吗喃) (卡馬西

For peer review only

For peer review only - http://bmjopen.bmj.com/site/about/guidelines.xhtml

	or quetiapine or citalopram or dextromethorphan or carbamazepine or mirtazapine or sertraline or moclobemide or trazodone or melatonin or ramelteon or methylphenidate).mp.	平 or 卡马平 or 卡巴氮平 or 卡巴马平) (米氮平) (舍曲林) (吗氯贝胺) (曲唑酮) (褪黑素 or 褪黑激素) (雷美替胺 or 拉米替隆) (哌甲酯 or 派醋甲酯 or 盐酸甲酯)
18	Movement Therapy	(运动 or 动作)
19	(Physical activit* or physical training).mp.	(运动 or 体育 or 体能) (活动 or 训练)
20	(social adj3 activit*).mp.	社交活动 or 社会活动
21	Psychotherapy	心理 (治疗 or 疗法)
22	(behavio?r* adj3 therap*).mp.	行为 (治疗 or 疗法)
23	Counseling	辅导 or 咨询
24	((Psychosocial or psycho social) adj3 (support or interven* or care)).ti,ab.	（社会心理 or 社交心理）（支援 or 治疗 or 干预 or 介入 or 照顾)
25	Alternative medicine	(替代 or 另类) (治疗 or 疗法 or 医学 or 医疗)
26	Chinese medicine	中医 or 中药
27	Acupuncture	针灸 or 针刺 or 电针
28	(herb* adj3 (tea or remedy or remedies or medicine*)).ti,ab.	草药 or 药草 or 药用植物 or 草本 or 茶疗
29	Gingko	银杏 or 白果
30	homeopathy	(顺势 or 同质 or 同种)
31	((music or art or aroma or light or photo or pet or pets) adj3 therap*).ti,ab.	(音乐 or 艺术 or 香薰 or 光照 or 光线 or 宠物 or 动物 or 舞蹈)
32	Massage	按摩 or 推拿
33	Mind Body Therapy	身心 or 心身 or 正念 or 冥想
34	Advance directives	预设医疗指示 or 预设指示 or 预前意愿 or 预先指示
35	(Advance? adj3 (care or medical or healthcare) adj3 plan*).mp.	(预设 or 预立) (护理计划 or 临终*计划 or 医疗决定)
36	(decision* adj3 (aid* or support)).mp.	决策援助 or 决策辅助 or 决策支持
37	Case Management	个案管理
38	(communicati* adj3 skill* adj3 training).mp.	沟通技巧 (训练 or 培训)

For peer review only - http://bmjopen.bmj.com/site/about/guidelines.xhtml

39	(dementia care adj3 map*).mp.	认知障碍症照顾图谱 or 老年痴呆症照顾图谱 or 失智症照顾测绘
40	((person* or patient*) adj3 cent* adj3 care).mp.	(以人为本 or 人本 or 以人为中心 or 病人为本) (照顾 or 照护 or 护理 or 治疗 or 医疗)
41	((caregiver or carer) adj3 educat*).mp.	(照顾者 or 家属 or 家庭 or 照护者 or 照料者) 教育
42	Support Groups	（支援 or 支持 or 互助）（小组 or 组）
43	Self-Help Techniques	自助法 or 自助*法 or 自助技巧 or 自救*
44	Social Support	社交支援/社交支持/互助组
45	Computer assisted diagnosis	电脑辅助诊断/计算机辅助诊断
46	Telemedicine	远程医疗 or 远距医疗 or 远距离医疗
47	Computer Assisted Therapy	电脑辅助治疗 or 计算机辅助治疗
48	Mobile Devices	移动设备 or 行动装置
49	((smart adj2 (phone* or device* or tablet*)) or smartphone*).mp.	智能 (手机 or 电话 or 平板电脑) or 智能* or 可穿戴*
50	cognitive aid	认知辅助/认知帮助
51	Reminder	提示 or 提醒
52	Robot	机器人
53	Animal Experiment	动物实验/动物试验

For peer review only

Box 1 Data to be extracted from included studies

General information

- Reviewers' name
- Date of data extraction
- Publication details and identification
- Sponsorship source
- Research site: places (city-level) where the trial was conducted
- Setting (e.g., hospital, care home, community)
- Study aim(s)
- Publication language: Chinese or English

Methods

- Study design
- List of all outcomes with instruments reported in the study

Population

- Inclusion criteria
- Exclusion criteria
- Group differences
- Clinical features (e.g., types of dementia, severity and duration of dementia)
- Baseline characteristics of participants in each study arm or overall participants: demographics (e.g., age, gender), socioeconomic status (e.g., education), clinical outcomes if any, number of participants

Intervention

- Description of the intervention(s) and comparator(s), including intervention name, treatment dose, duration, components and how it was delivered.
- Intervention type (e.g., pharmaceutical intervention, traditional Chinese medicine, non-pharmacological treatment and multicomponent interventions).

Outcomes

- Outcome name including the name of each outcome of interest and how it was measured (instruments used)
- Outcome type and reported format. The components of reporting effect measures are: (1) the effect measure itself (e.g., change from baseline), (2) a measure of its variance (e.g., the standard deviation, or the 95% CI), (3) the number of participants in the study arm (N).
- Scale and direction of effect

Results

- Results of outcomes reported in the original study at each time point.

Risk of bias information

- Judgements based on the criteria of the RoB 2.

For peer review only - http://bmjopen.bmj.com/site/about/guidelines.xhtml

Reporting checklist for protocol of a systematic review.

Based on the PRISMA-P guidelines.

Instructions to authors

Complete this checklist by entering the page numbers from your manuscript where readers will find each of the items listed below.

Your article may not currently address all the items on the checklist. Please modify your text to include the missing information. If you are certain that an item does not apply, please write "n/a" and provide a short explanation.

Upload your completed checklist as an extra file when you submit to a journal.

In your methods section, say that you used the PRISMA-Preporting guidelines, and cite them as:

Moher D, Shamseer L, Clarke M, Ghersi D, Liberati A, Petticrew M, Shekelle P, Stewart LA. Preferred Reporting Items for Systematic Review and Meta-Analysis Protocols (PRISMA-P) 2015 statement. Syst Rev. 2015;4(1):1.

For peer review only

		Reporting Item	Page Number
**Title**			
Identification	#1a	Identify the report as a protocol of a systematic review	1
Update	#1b	If the protocol is for an update of a previous systematic review, identify as such	n/a

**Registration**			
	#2	If registered, provide the name of the registry (such as PROSPERO) and registration number	4
**Authors**			
Contact	#3a	Provide name, institutional affiliation, e-mail address of all protocol authors; provide physical mailing address of corresponding author	1-2
Contribution	#3b	Describe contributions of protocol authors and identify the guarantor of the review	2
**Amendments**			
	#4	If the protocol represents an amendment of a previously completed or published protocol, identify as such and list changes; otherwise, state plan for documenting important protocol amendments	n/a
**Support**			
Sources	#5a	Indicate sources of financial or other support for the review	2
Sponsor	#5b	Provide name for the review funder and / or sponsor	2
Role of sponsor or funder	#5c	Describe roles of funder(s), sponsor(s), and / or institution(s), if any, in developing the protocol	2
**Introduction**			

For peer review only

Rationale	#6	Describe the rationale for the review in the context of what is already known	5-7
Objectives	#7	Provide an explicit statement of the question(s) the review will address with reference to participants, interventions, comparators, and outcomes (PICO)	7
**Methods**			
Eligibility criteria	#8	Specify the study characteristics (such as PICO, study design, setting, time frame) and report characteristics (such as years considered, language, publication status) to be used as criteria for eligibility for the review	7-11
Information sources	#9	Describe all intended information sources (such as electronic databases, contact with study authors, trial registers or other grey literature sources) with planned dates of coverage	11-12
Search strategy	#10	Present draft of search strategy to be used for at least one electronic database, including planned limits, such that it could be repeated	12-13
Study records - data management	#11a	Describe the mechanism(s) that will be used to manage records and data throughout the review	13
Study records - selection process	#11b	State the process that will be used for selecting studies (such as two independent reviewers) through each phase of the review (that is, screening, eligibility and inclusion in meta-analysis)	13-14

For peer review only

Study records - data collection process	#11c	Describe planned method of extracting data from reports (such as piloting forms, done independently, in duplicate), any processes for obtaining and confirming data from investigators	14-15
Data items	#12	List and define all variables for which data will be sought (such as PICO items, funding sources), any pre-planned data assumptions and simplifications	15&30
Outcomes and prioritization	#13	List and define all outcomes for which data will be sought, including prioritization of main and additional outcomes, with rationale	15-16
Risk of bias in individual studies	#14	Describe anticipated methods for assessing risk of bias of individual studies, including whether this will be done at the outcome or study level, or both; state how this information will be used in data synthesis	16-17
Data synthesis	#15a	Describe criteria under which study data will be quantitatively synthesised	18-19
Data synthesis	#15b	If data are appropriate for quantitative synthesis, describe planned summary measures, methods of handling data and methods of combining data from studies, including any planned exploration of consistency (such as I2, Kendall's τ)	18-19
Data synthesis	#15c	Describe any proposed additional analyses (such as sensitivity or subgroup analyses, meta-regression)	19

For peer review only

Data synthesis	#15d	If quantitative synthesis is not appropriate, describe the type of summary planned	17
Meta-bias(es)	#16	Specify any planned assessment of meta-bias(es) (such as publication bias across studies, selective reporting within studies)	19-20
Confidence in cumulative evidence	#17	Describe how the strength of the body of evidence will be assessed (such as GRADE)	20

The PRISMA-P checklist is distributed under the terms of the Creative Commons Attribution License CC-BY 4.0. This checklist was completed on 02. December 2020 using https://www.goodreports.org/, a tool made by the EQUATOR Network in collaboration with Penelope.ai

For peer review only
